# Beyond Histocompatibility: Donor Tissue Immune States and Their Role in Heart Transplantation

**DOI:** 10.3390/ijms27208985

**Published:** 2026-10-10

**Authors:** Tomasz Urbanowicz, Mariusz Kowalewski, Piotr Suwalski, Giuseppe Maria Raffa

**Affiliations:** 1 Cardiac Surgery and Transplantology Department, Poznan University of Medical Sciences, ½ Długa, 61-848 Poznań, Poland; 2 Thoracic Research Center, Collegium Medicum, Nicolaus Copernicus University, 85-796 Bydgoszcz, Poland; 3 Department of Precision Medicine in Medical Surgical and Critical Area (Me.Pre.C.C.), University of Palermo, 90134 Palermo, Italy; giuseppe.raffa78@gmail.com; 4 Department of Cardiac Surgery, Center of Postgraduate Medical Education, Central Clinical Hospital of the Ministry of Interior, 02-507 Warszawa, Poland; piotr.suwalski@pimmswia.gov.pl; 5 Department of Research, IRCCS-ISMETT (Mediterranean Institute for Transplantation and Specialized Therapies), 90127 Palermo, Italy

**Keywords:** heart transplantation, donor tissue, tissue-resident immunity, trained immunity, macrophages, immune memory, donor–recipient interaction, cardiac allograft vasculopathy, single-cell analysis, spatial transcriptomics

## Abstract

Heart transplantation transfers not only donor alloantigens and functional myocardium but also a pre-existing tissue environment shaped by resident immune, vascular, stromal, and parenchymal populations. This review examines the hypothesis that the biological state of donor tissue influences subsequent recipient immunity and contributes to heterogeneity in allograft outcomes. Donor-resident macrophages, endothelial cells, fibroblasts, extracellular matrix, and tissue-resident lymphocytes constitute an immunologically active myocardial niche that encounters ischemia–reperfusion injury and recipient immune effectors immediately after implantation. Experimental and human studies demonstrate the dynamic coexistence and replacement of donor- and recipient-derived immune populations, with recipient cells acquiring phenotypes within the graft microenvironment. We distinguish transient donor-tissue activation from genuine tissue immune memory, which would require persistence of an exposure-induced molecular or functional state and an altered response to a subsequent challenge. Potential mechanisms include trained innate immunity, epigenetic and metabolic reprogramming, endothelial inflammatory memory, and persistent stromal–immune interactions. We propose that allograft biology be investigated at the level of the donor–recipient immune pair and outline longitudinal single-cell, spatial, and cell-of-origin approaches to test this concept in human heart transplantation.

## 1. Introduction

Heart transplantation remains the treatment of choice for selected patients with end-stage heart failure [1]. Yet long-term graft survival remains limited by immune-mediated injury and cardiac allograft vasculopathy (CAV) [2,3]. Major advances in donor–recipient matching, immunosuppressive therapy, surveillance of donor-specific antibodies (DSA), and the diagnosis and treatment of acute rejection have substantially improved early outcomes [4]. Considerable differences in graft behavior persist among recipients who appear to have comparable immunological risk according to conventional clinical criteria [5,6]. Some allografts remain functionally stable for many years, whereas others develop early inflammatory injury, recurrent rejection, fibrosis, or accelerated CAV. The biological mechanisms underlying this variability are only partly understood.

Transplant immunology has traditionally approached this problem primarily from the recipient’s perspective. The donor organ presents alloantigens that the recipient’s immune system recognizes and responds to, ultimately determining whether rejection or tolerance occurs [7,8]. This framework has placed HLA compatibility, preformed and de novo DSA, T-cell activation, and the intensity of immunosuppression at the center of post-transplant risk assessment. Although this model remains fundamental to clinical practice, it does not fully account for the immunological state of the donor tissue in which alloimmune recognition subsequently occurs.

The donor heart is not an immunologically passive structure composed solely of cardiomyocytes and vascular tissue. At the time of procurement, it contains heterogeneous populations of resident macrophages, dendritic cells, endothelial cells, fibroblasts, lymphocyte subsets, and other stromal and immune cells organized within distinct myocardial and perivascular niches [9,10]. These populations have developed within the donor and acquired tissue-specific functional states before transplantation. The relevant question is therefore not simply what cells are transferred with the graft, but whether their pre-existing functional states influence the subsequent response to ischemia–reperfusion and recipient immunity.

This question has become particularly relevant with the growing understanding of tissue-resident immunity. Tissue macrophages and other resident immune populations are increasingly recognized as locally adapted cells whose transcriptional, metabolic, and epigenetic programs differ from those of their circulating counterparts [11,12]. In other biological settings, previous inflammatory or metabolic exposure can produce durable changes in innate immune responsiveness, providing the basis for the concepts of trained immunity and innate immune memory [13,14,15]. Whether comparable programs are established within the human donor myocardium, persist until transplantation, and remain functionally relevant after transfer into a new host is unknown. This unresolved question forms a central hypothesis of the present review.

Transplantation provides a unique biological setting in which such pre-existing tissue states could become functionally relevant. Procurement, preservation, and reperfusion impose an intense sequence of injury signals that activate donor endothelium and resident innate immune cells before a mature recipient alloimmune response is established. The resulting inflammatory response may therefore depend not only on the magnitude of transplant-associated injury but also on the functional state of the donor-resident cells that encounter it.

The immune composition of the transplanted heart is not static after implantation. Donor-derived resident cells may persist for variable periods, whereas recipient-derived leukocytes progressively enter the graft and contribute to tissue remodeling [16,17]. The resulting allograft becomes a dynamic interface between a donor-derived tissue environment and an independently conditioned recipient immune system. Early interactions among donor-resident macrophages, vascular endothelium, stromal cells, and infiltrating recipient immune populations could influence alloimmune priming, the resolution or persistence of inflammation, extracellular matrix remodeling, and, ultimately, the development of chronic graft dysfunction [18].

Recent methodological advances now make this interaction experimentally accessible. Single-cell and spatial approaches can resolve the identity, functional state, and anatomical localization of donor-resident and recipient-derived populations within the cardiac allograft [19,20,21]. Epigenetic profiling, proteomics, metabolomics, and high-dimensional immune phenotyping may further identify persistent molecular programs that are not apparent from conventional histological assessment [22,23,24]. When combined with methods capable of determining cellular origin, longitudinal sampling could establish whether donor-derived immune states persist after implantation, whether recipient cells acquire distinct phenotypes within the donor tissue niche, and whether these cellular transitions are associated with rejection, inflammatory resolution, fibrosis, or CAV. Such studies would allow the donor–recipient interaction itself, rather than either compartment in isolation, to become the unit of biological investigation.

Because direct evidence for exposure-induced immune memory in the human donor heart remains limited, we distinguish throughout this review among five levels of evidence: (1) human heart transplantation; (2) experimental cardiac transplantation; (3) non-transplant cardiac biology; (4) other solid-organ or hematopoietic transplantation; and (5) extrapolative mechanistic evidence from non-cardiac systems. Evidence from the latter categories is used to establish biological plausibility and generate testable hypotheses rather than as proof that donor-heart immune memory exists in humans.

This narrative review was informed by a structured literature search of PubMed/MEDLINE for studies relevant to donor-resident immunity, cardiac transplantation, trained innate immunity, donor–recipient cellular chimerism, endothelial and stromal immune interactions, ischemia–reperfusion injury, and single-cell or spatial profiling of cardiac allografts. Searches combined terms related to “heart transplantation”, “cardiac allograft”, “donor macrophage”, “tissue-resident macrophage”, “trained immunity”, “innate immune memory”, “donor–recipient chimerism”, “endothelium”, “fibroblast”, “extracellular matrix”, “single-cell RNA sequencing”, and “spatial transcriptomics”. Priority was given to studies on human heart transplantation and experimental cardiac transplantation models. Evidence from non-transplant cardiac biology, other solid-organ transplantation, and non-cardiac mechanistic studies was included when directly relevant to biological plausibility and is identified as indirect or extrapolative evidence. Reference lists of relevant reviews and primary studies were additionally screened for pertinent publications. Because the purpose of this article is conceptual synthesis rather than the aggregation of quantitative evidence, the review was not conducted as a formal systematic review or meta-analysis. The final literature search was updated through September 2026.

In this review, we examine the hypothesis that cardiac transplantation transfers not only donor alloantigens and functional myocardium but also an established tissue immune environment that influences the subsequent recipient response. We focus on three related questions: whether donor-resident immune, endothelial, and stromal populations carry functionally relevant states into transplantation; how these populations interact with recipient-derived cells during reperfusion and subsequent alloimmune activation; and whether this interaction contributes to inflammatory resolution, rejection, fibrosis, and CAV. Particular attention is given to tissue-resident macrophages, endothelial–immune and stromal–immune interactions, cellular replacement after transplantation, and the possibility of persistent innate immune programming. We finally define the experimental evidence required to establish donor tissue immune memory in humans and propose a framework for studying the donor–recipient immune pair as a determinant of allograft biology.

## 2. The Donor Heart as an Immunologically Active Organ

**Figure 1:**
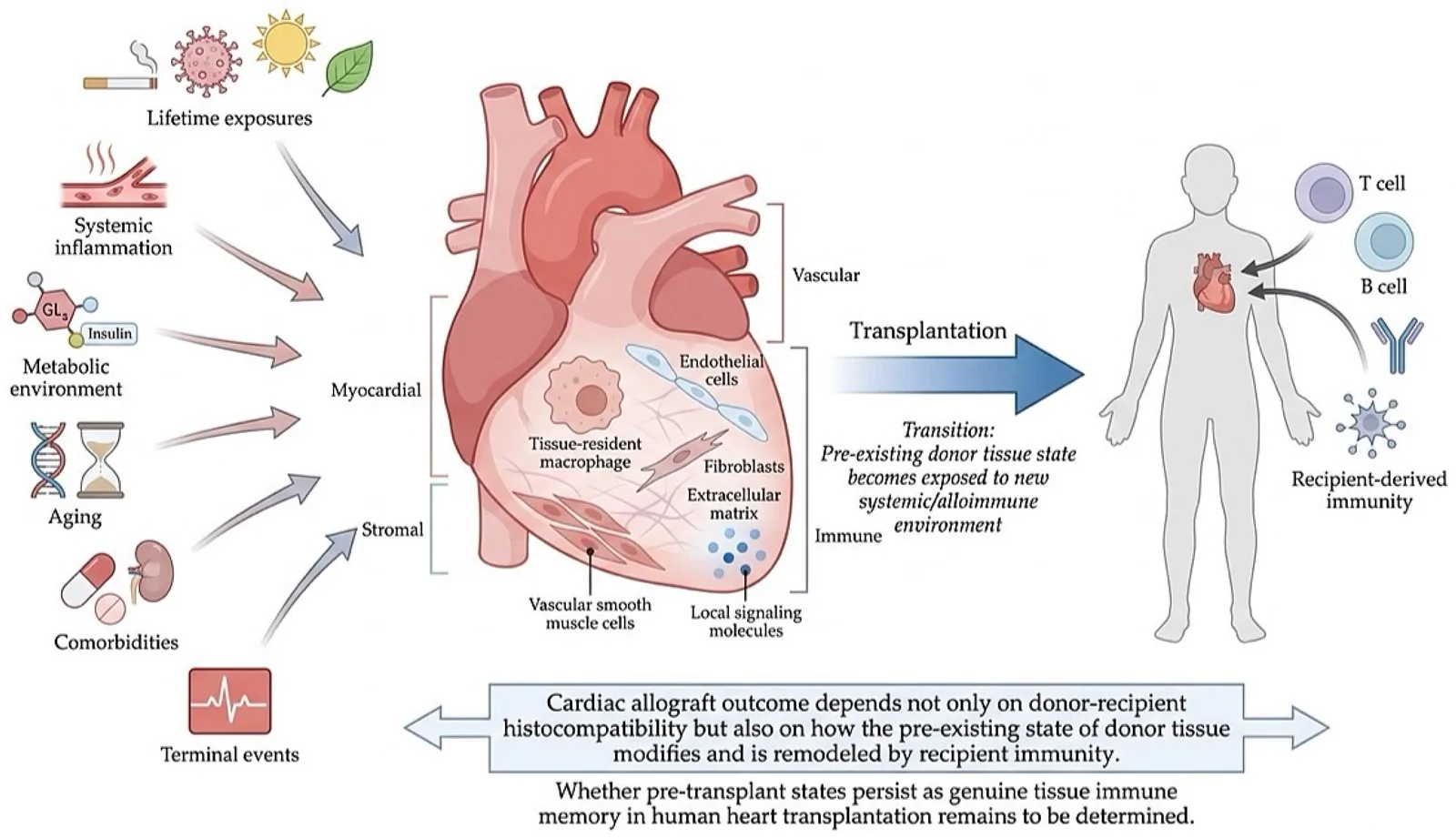
The Donor Heart as an Immunologically Active Organ. Lifetime biological exposures shape the immune, vascular, stromal, and parenchymal state of the donor heart before procurement. Transplantation transfers this pre-existing tissue environment into the recipient, where it encounters ischemia–reperfusion injury and recipient-derived cellular and humoral immunity. Subsequent allograft reflects dynamic interaction between the pre-existing donor-tissue state and recipient immunity. Abbreviations: B cell, B lymphocyte; GLs, glucose/lipid-related metabolic factors; T cell, T lymphocyte. Created by Urbanowicz T. with www.figurelabs.com, on 13 September 2026, certificate ID: FL-PUB-20260913-RS5XYW.

The conventional description of cardiac transplantation begins with recognition of donor alloantigens by the recipient immune system. Although fundamental to the biology of rejection, this framework can reduce the graft conceptually to a source of alloantigen and a target of recipient immune injury. The myocardium is instead an organized multicellular tissue in which immune, vascular, stromal, and parenchymal populations cooperate in homeostasis and respond collectively to injury [9,25]. This cellular network was already established before procurement. Transplantation transfers not only functioning myocardium and donor HLA but also an intact donor-derived tissue environment that actively participates in the earliest post-transplant response (Figure 1).

The principal cellular components of this donor-derived tissue environment, their persistence after transplantation, and their potential contribution to allograft biology are summarized in Table 1.

Cardiac macrophages constitute a heterogeneous population whose functions extend well beyond phagocytosis and inflammatory defense [35]. Under physiological conditions, they participate in the clearance of cellular debris, the maintenance of tissue integrity, vascular homeostasis, extracellular matrix turnover, and repair [36]. Experimental work has further implicated specialized macrophage populations in myocardial conduction and coronary vascular adaptation, emphasizing the extent to which resident immunity is integrated into normal cardiac physiology [37,38]. Importantly, these cells are not functionally interchangeable with monocytes recruited from the circulation. Ontogeny, tissue residence, local self-renewal, and continuous exposure to myocardial signals generate functional states that distinguish resident cardiac macrophages from newly recruited circulating monocytes.

The donor heart does not enter the recipient as an immunological void that is subsequently populated by recipient leukocytes [16]. Donor-derived resident macrophages and other immune populations are present at reperfusion, while recipient monocytes and other circulating leukocytes progressively enter the graft and acquire tissue-adapted phenotypes [17]. The early allograft is therefore a cellular chimera in which donor-resident and recipient-derived immune populations coexist within the same myocardial environment. Importantly, this period coincides with ischemia–reperfusion injury, complement and endothelial activation, DAMP (damage-associated molecular pattern) release, and the initiation of alloimmune recognition [17].

The immunological identity of the graft, however, cannot be reduced to its leukocyte compartment. Donor endothelial cells occupy a particularly important position because they form the interface between the transplanted organ and the recipient circulation [39]. They are simultaneously targets of alloimmune recognition and active sensors of the local environment [40]. Hypoxia, reactive oxygen species, complement, cytokines, disturbed shear stress, and metabolic injury can rapidly shift endothelial cells from a homeostatic toward an activated phenotype, promoting adhesion and transmigration of recipient leukocytes and amplifying local inflammatory signaling [41]. The endothelial compartment, therefore, provides a mechanistic bridge between non-immune graft injury and alloimmunity. Ischemia–reperfusion injury and rejection should therefore not be regarded as entirely separate biological processes when the former modifies the cellular interface on which the latter develops [42].

Cardiac fibroblasts’ traditional designation as structural cells responsible for extracellular matrix production substantially underestimates their role in tissue immunity [43]. Fibroblasts sense inflammatory mediators, mechanical stress, hypoxia, and signals released by injured cardiomyocytes. In response, they can produce chemokines, cytokines, growth factors, and matrix components that influence leukocyte recruitment, macrophage polarization, and persistence of local inflammation [44,45]. Conversely, macrophage- and lymphocyte-derived signals regulate fibroblast activation and differentiation [46]. Endothelial cells, macrophages, and fibroblasts consequently form interconnected signaling circuits that determine whether an acute inflammatory response resolves or evolves into persistent inflammation and fibrosis [47]. The relevant biological unit is therefore not an isolated leukocyte population but a multicellular myocardial niche in which immune and structural cells continuously exchange information. These interactions are particularly relevant to transplantation because recipient-derived immune cells enter a myocardial niche whose endothelial, stromal, extracellular matrix, and parenchymal architecture remain predominantly donor-derived [41].

The biological influence of donor-resident cells may also outlast their physical persistence. Transient donor-derived populations can modify endothelial phenotype, fibroblast activation, extracellular matrix organization, and the recruitment or differentiation of recipient cells [48,49]. Cellular replacement does not necessarily restore an immunologically neutral tissue environment. Rather, recipient cells enter and differentiate within an architecture that remains largely donor-derived. The donor heart should consequently be regarded as an immunologically active organ whose resident cells and structural compartments help shape the environment in which recipient immunity subsequently operates. This perspective does not diminish the central role of recipient adaptive immunity; it defines the tissue context in which that response occurs.

Throughout the remainder of this review, evidence that donor-resident cells influence post-transplant inflammation is interpreted as evidence of donor-tissue functionality, whereas the term donor-heart immune memory is reserved for the more stringent and currently unproven scenario in which an antecedent exposure generates a persistent cellular program that modifies the response to a subsequent transplant-associated challenge.

## 3. Tissue-Resident Immune Memory: Can the Heart Remember Previous Injury?

Immune memory has traditionally been considered a property of adaptive immunity [50,51]. This definition has broadened in light of evidence that innate immune cells can retain functional consequences of prior exposure. Following infection, inflammatory stimulation, or metabolic stress, monocytes and macrophages may respond differently to a subsequent challenge even after the initiating stimulus has been removed [52]. Such persistence can be mediated by coordinated epigenetic, transcriptional, and metabolic reprogramming and provides the mechanistic basis for trained immunity [50,53]. Whether comparable persistent programs exist within resident cells of the human donor heart, survive the transplantation process, and modify subsequent allograft responses remains unknown.

**Figure 2:**
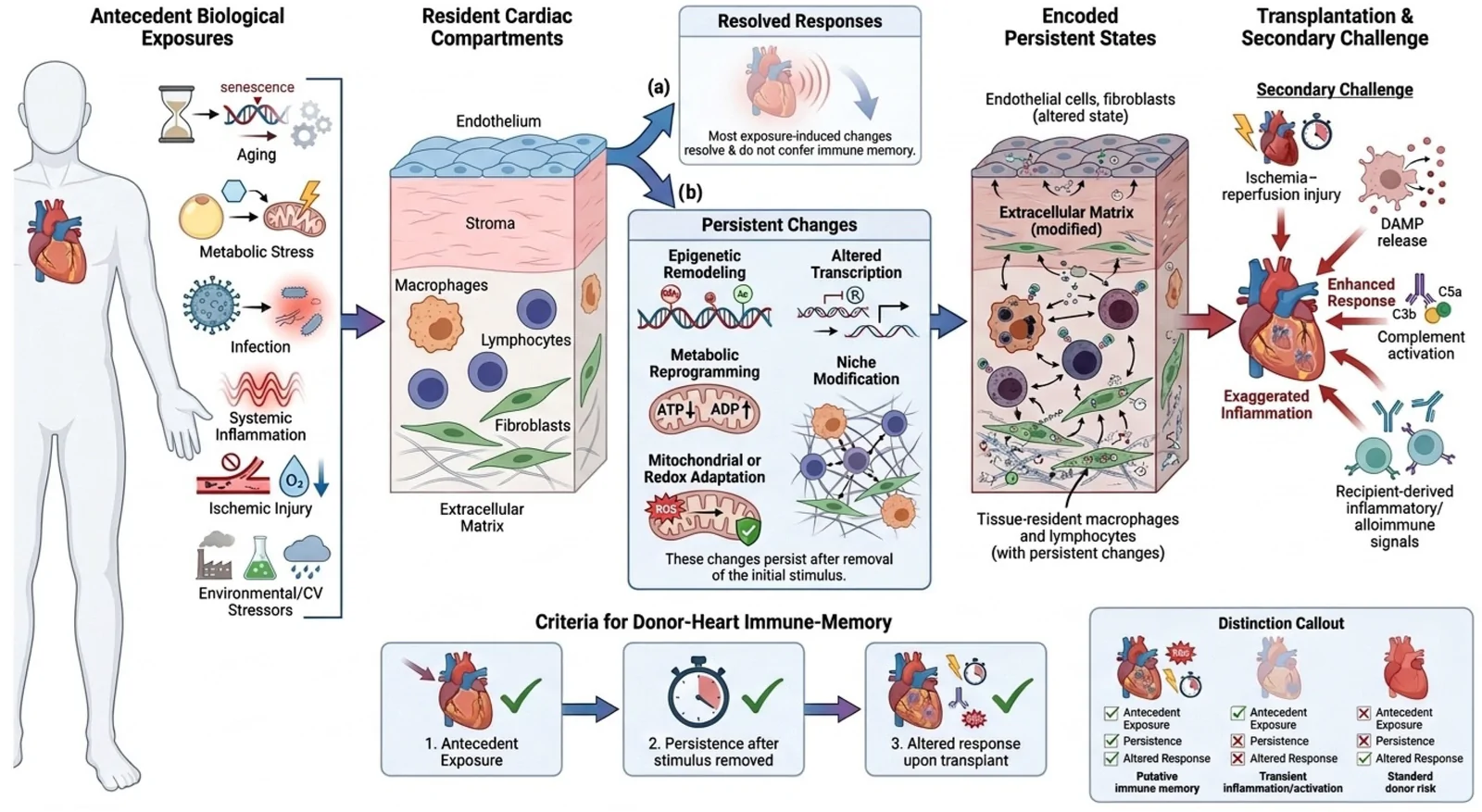
From Previous Donor Exposure to Persistent Tissue Immune State: A Testable Model of Donor-Heart Immune Memory. Antecedent biological exposures may resolve or generate persistent cellular or tissue alterations that influence the response to transplantation-associated secondary challenge. Cell-intrinsic immune memory requires persistent exposure-induced programming and altered secondary responsiveness, whereas structural or matrisomal persistence represents a tissue-level imprint rather than cellular immune memory. Abbreviations: ADP, adenosine diphosphate; ATP, adenosine triphosphate; C3b, complement component 3b; C5a, complement component 5a; CV, cardiovascular; DAMPs, damage-associated molecular patterns; ROS, reactive oxygen species. Created by Urbanowicz T. with www.figurelabs.com, on 13 September 2026, certificate ID: FL-PUB-20260913-19IIYF.

The relevance of antecedent donor exposure to the present framework depends on whether it produces a persistent change in the immune or stromal state of the myocardium. Donor hearts may differ in this respect, but exposure itself should not be equated with tissue immune memory [54,55]. Some of these influences may be transient, whereas others may leave more durable changes in resident immune cells, endothelium, and the stromal compartment [56]. The central issue is therefore persistence rather than exposure itself. Donor hearts inevitably differ in their previous biological environments, but such differences are relevant to an immune-memory hypothesis only if they leave a cellular or tissue alteration that remains detectable after the initiating exposure has resolved. The relevant question is whether such persistent states modify the graft’s response to subsequent challenges, particularly ischemia–reperfusion and recipient immune activation (Figure 2).

Cell-intrinsic immune memory should be distinguished from tissue-level structural persistence. The former requires durable exposure-induced epigenetic, transcriptional, or metabolic reprogramming within surviving cells that alters their response to a subsequent challenge, whereas persistent endothelial, stromal, or extracellular-matrix remodeling represents a microenvironmental or matrisomal imprint that may shape the behavior of subsequently recruited recipient-derived cells without itself constituting cellular immune memory [53,57,58]. These mechanisms may coexist but should not be considered synonymous.

Trained immunity provides one mechanistic framework through which such persistence could occur. Innate immune training is associated with durable changes in chromatin accessibility, transcriptional regulation, and cellular metabolism that modify the response to later stimulation [59]. These mechanisms can maintain altered cellular responsiveness after the initiating signal has disappeared. Importantly, this state is neither constitutive inflammation nor conventional antigen-specific memory; it represents a persistent change in the threshold or character of a subsequent innate response [60,61].

Transplantation provides a particularly informative setting in which to test this hypothesis because reperfusion constitutes a defined secondary challenge. If persistent donor-derived tissue states are biologically relevant, their consequences should become apparent when resident cells encounter ischemia–reperfusion-associated DAMPs, complement activation, and recipient-derived inflammatory signals [62,63]. The same transplant-associated insult could elicit different responses depending on the donor tissue’s pre-existing functional state.

Epigenetic remodeling, altered mitochondrial function, disrupted redox signaling, and impaired nitric oxide homeostasis are potential mechanisms by which prior exposure may influence subsequent endothelial behavior [64,65]. Previous inflammatory or metabolic exposure can produce persistent changes in endothelial responsiveness, although it remains uncertain whether such states constitute transplant-relevant tissue memory. This question is particularly relevant in heart transplantation because graft endothelium is directly exposed to recipient antibodies, complement, circulating leukocytes, and inflammatory mediators. A persistent endothelial state could therefore modify the context in which recipient alloimmunity is initiated or amplified.

Persistent tissue states may also be encoded outside classical immune populations. Fibroblasts can retain altered phenotypes after injury, while changes in extracellular matrix composition can modify subsequent immune cell localization and behavior [66]. Interactions among fibroblasts, macrophages, and endothelial cells may therefore provide a mechanism by which the consequences of an earlier exposure persist within the tissue niche rather than within a single cellular population [67].

Trained innate immunity requires an exposure-induced epigenetic, transcriptional, or metabolic program that persists within a cell and alters its response to subsequent challenges [68,69]. In contrast, a modified extracellular matrix does not itself constitute immune memory but may represent a durable matrisomal or microenvironmental change, including changes in matrix composition, stiffness, spatial organization, or endothelial–stromal architecture. Such alterations can shape the recruitment, differentiation, and inflammatory behavior of recipient-derived cells [70]. Thus, cellular memory preserves information within surviving cells, whereas structural persistence preserves it within the tissue environment encountered by subsequent cells. Both may influence post-transplant biology but are mechanistically distinct.

Throughout this review, these terms are used according to the operational definitions summarized in Table 2. To avoid conceptual overlap, we use “biological history” only as a descriptive term for antecedent donor exposures, “conditioning” for exposure-related modification of the tissue state irrespective of persistence, “persistent donor state” for an alteration demonstrable after withdrawal of the initiating stimulus, and “immune memory” only when such persistence is accompanied by an altered response to a defined subsequent challenge. In particular, a baseline donor phenotype or persistent tissue alteration should not be interpreted as immune memory unless it persists after the initiating exposure has resolved and is accompanied by an altered response to a subsequent challenge.

Progressive replacement of donor leukocytes by recipient-derived populations does not necessarily eliminate the biological consequences of the pre-existing donor tissue state. If persistence depended exclusively on the long-term survival of donor macrophages, its influence would diminish as these populations disappeared. Such biological continuity does not necessarily require the persistence of the original donor immune cell population. Donor-resident cells may modify endothelial activation, stromal behavior, extracellular-matrix organization, and the recruitment or differentiation of recipient monocytes during the early post-transplant period [16]. Recipient-derived cells subsequently entering this environment may therefore encounter a niche partly shaped before their arrival. Cellular replacement need not restore an immunologically neutral tissue state. The principal donor exposures, candidate tissue compartments in which their effects may persist, and mechanisms through which they could modify the response to transplantation are summarized in Table 3.

Persistent inflammation is not synonymous with trained immunity, and the presence of an activated phenotype at procurement does not demonstrate that the tissue retains information from a previous resolved exposure [69]. Similarly, associations between donor age, metabolic disease, or cause of death and post-transplant outcome cannot establish tissue memory [83,84]. A more rigorous definition requires three elements: an antecedent exposure, persistence of a molecular or functional alteration after that exposure has ceased, and a demonstrably altered response to a later stimulus.

Evidence satisfying these criteria in the human donor heart remains limited. Much of the necessary biology could not previously be resolved in human myocardial samples. Bulk tissue analysis cannot reliably distinguish a stable program within a small resident population from a transient change in cellular composition. Nor can it establish whether the relevant signal originates from donor-derived macrophages, endothelium, fibroblasts, or infiltrating recipient cells.

Single-cell and spatial approaches now make this distinction increasingly accessible. In transplantation, these approaches will be most informative when donor tissue obtained before implantation can be linked to early and late post-transplant samples and to clinically defined graft phenotypes. Such longitudinal studies could test whether molecular states present in the donor organ survive transplantation directly or indirectly, whether they influence the recruitment and differentiation of recipient cells, and whether they provide information beyond established donor and recipient risk factors. A particularly informative design would pair pre-implant donor myocardial profiling with cell-of-origin-resolved analysis of early and late allograft samples. Such studies could distinguish persistence of donor-derived programs from changes caused by recipient-cell infiltration and determine whether a pre-existing donor state modifies the subsequent response to reperfusion or alloimmune injury.

We propose that persistent consequences of the donor heart’s biological history should be investigated at two mechanistically distinct levels. The first is cell-intrinsic memory, in which surviving donor-derived immune, endothelial, or stromal cells retain exposure-induced epigenetic, transcriptional, or metabolic programs that modify their response to a subsequent challenge [85]. The second is tissue-level structural persistence, in which antecedent injury leaves a durable endothelial, stromal, extracellular-matrix, or spatial imprint that subsequently modifies the behavior of recipient-derived cells without itself constituting cellular immune memory [86,87]. These mechanisms may coexist and interact, but demonstrating one does not establish the other. Accordingly, claims of donor-heart immune memory should require evidence of persistent cellular programming and altered secondary responsiveness, whereas persistent matrix or tissue architecture should be described as a microenvironmental or matrisomal imprint unless an active cellular memory mechanism is demonstrated.

## 4. Donor Tissue State and Recipient Immunity: An Interactive Model of Allograft Biology

Transplantation places an immunologically active donor tissue within a recipient immune system that has developed independently of the graft [88]. Conventional histocompatibility remains fundamental to this interaction: donor alloantigens are recognized by recipient lymphocytes, and the magnitude and regulation of the response influence whether rejection or accommodation occurs [89]. Histocompatibility alone, however, does not describe the cellular context in which alloantigen recognition occurs. At implantation, donor-resident immune cells, vascular endothelium, and stromal populations encounter recipient leukocytes and circulating immune mediators for the first time [90]. The early allograft represents a site of interaction between graft-resident and recipient-derived immune mechanisms rather than a passive target of recipient immunity.

Established pathways of allorecognition already involve contributions from both donor- and recipient-derived compartments [91]. In direct allorecognition, recipient T cells recognize intact donor MHC–peptide complexes, classically presented by donor antigen-presenting cells carried within the graft. Indirect allorecognition follows processing of donor antigens by recipient antigen-presenting cells [92], whereas semidirect allorecognition occurs when recipient antigen-presenting cells acquire intact donor MHC complexes, including through extracellular-vesicle transfer [93]. The relative contribution of these pathways changes over time after transplantation [83]. Semidirect recognition is particularly relevant to the present framework because it demonstrates that recipient cells can retain and present intact donor-derived molecular structures [83].

The biology of donor-resident macrophages provides a particularly instructive example. Experimental cardiac transplantation has shown that donor macrophages are not passive passenger leukocytes [94]. Distinct CCR2 (chemokine receptor-2)—positive and CCR2-negative macrophage populations remain within transplanted hearts and coexist with infiltrating recipient monocytes and monocyte-derived macrophages [95]. In a murine model of cardiac allograft rejection, depletion of donor CCR2-positive macrophages prolonged graft survival, whereas depletion of CCR2-negative macrophages had the opposite effect [96]. Disruption of MYD88 (myeloid differentiation primary response 88) signaling specifically in donor macrophages reduced recruitment of antigen-presenting cells, impaired subsequent T-cell activation, and prolonged allograft survival [97]. The state of the donor innate immune compartment can therefore influence the magnitude of the recipient adaptive response rather than simply accompany it.

These findings establish the functional importance of the donor-resident innate immune compartment but should not be interpreted as evidence of donor-cell immune memory. Demonstration of memory would additionally require evidence that the relevant donor cells had been modified by an antecedent exposure, retained that program after resolution of the initiating stimulus, and exhibited an altered response to a subsequent challenge [57,68,97]. The experimental transplantation studies discussed here provide mechanistic support for donor-cell functionality, while the proposed extension to exposure-induced donor-cell memory remains hypothetical.

This interaction begins before conventional alloimmune rejection is established. In transplantation-associated ischemia–reperfusion injury, heart-resident CCR2-positive macrophages respond to tissue damage through TLR9 (Toll-like receptor-9)—MYD88—dependent signaling and regulate neutrophil recruitment through CXCL5 (C-X-C chemokine Ligand 5) [98]. The first recipient leukocytes entering the reperfused graft are therefore not responding to an immunologically inert myocardial environment. Their recruitment is, at least partly, orchestrated by cells already present in the donor heart. The distinction between donor and recipient immunity is consequently blurred from the first hours after implantation: donor-resident cells interpret graft injury, and help determine which recipient cells enter the organ and under what inflammatory conditions [57].

These observations have implications beyond acute inflammation. The macrophage compartment of the cardiac allograft undergoes substantial reconstruction after transplantation. Human single-nucleus transcriptomic studies have provided important evidence in this regard. Donor-derived tissue-resident macrophages accompany the transplanted heart but progressively disappear, while recipient monocytes enter the graft within days and generate new macrophage populations. Recipient-derived macrophages may acquire some features of the resident population they replace, yet they do not fully recapitulate its transcriptional state [16]. In human cardiac allografts, one recipient macrophage population resembling donor-resident macrophages lacked characteristic reparative phagocytic programs and instead expressed a more profibrotic signature [98]. It suggests that repopulation of the graft by recipient cells is not simply a change in cellular provenance. The recipient immune system enters a donor-derived tissue environment and develops within it, but the resulting cells are neither straightforward extensions of circulating recipient monocytes nor exact replacements for the original donor-resident populations. Their phenotype is generated within the transplanted organ.

The donor compartment initially influences recipient-cell recruitment and activation, while incoming recipient cells progressively modify the cellular composition and signaling environment of the graft [99]. These interactions occur within a tissue architecture that remains substantially graft-derived, although the extent of donor–recipient chimerism varies among cellular compartments [100]. The immune phenotype of the mature allograft is consequently neither purely donor nor purely recipient [6]. Unlike donor leukocytes, which may diminish substantially with time, the vascular endothelium remains an enduring donor-derived interface with recipient blood [101]. Structural and parenchymal components provide the local environment in which predominantly recipient-derived immune populations operate. The mature allograft is therefore a cellular mosaic in which graft-derived tissue compartments coexist and interact with progressively recruited recipient-derived populations [102].

This provides a plausible mechanism by which a donor’s biological history could modify the consequences of a given degree of histocompatibility. HLA mismatch describes antigenic disparity, but not the inflammatory context in which that disparity is presented. Two grafts with comparable HLA relationships to their recipients may differ substantially in endothelial senescence, mitochondrial reserve, basal inflammatory signaling, or susceptibility to complement-mediated injury. Conversely, two recipients with apparently comparable sensitization may respond differently if their circulating myeloid and lymphoid compartments have been conditioned by different biological histories.

Recipient immunity is also heterogeneous at the time of transplantation. Advanced heart failure is accompanied by systemic inflammatory and neurohumoral abnormalities, while sensitization may result from previous transfusion, surgery, pregnancy, or mechanical circulatory support [103,104]. Aging further modifies adaptive and innate immune function through immunosenescence and remodeling of hematopoiesis [105]. Clonal hematopoiesis may provide an additional source of heterogeneity because selected driver mutations are associated with altered myeloid inflammatory signaling, including inflammasome-dependent pathways [106]. These factors are relevant not because they constitute a single recipient’s “immune history”, but because they may alter the functional state of the cells and antibodies that subsequently encounter the graft. Previous sensitization also determines the repertoire of antigen-specific humoral and cellular memory available at the time of transplantation.

These observations suggest that donor and recipient characteristics may interact rather than contribute independently to allograft biology. The effect of a graft-resident inflammatory state may depend on the responsiveness of the recipient cells encountering it, just as the consequences of recipient alloimmune activation may depend on the cellular context within the graft. The clinically relevant biological unit may therefore be the donor–recipient immune pair rather than either compartment considered independently. This remains a hypothesis, but one that can now be tested directly using paired donor and recipient profiling and longitudinal analysis of the allograft.

**Figure 3:**
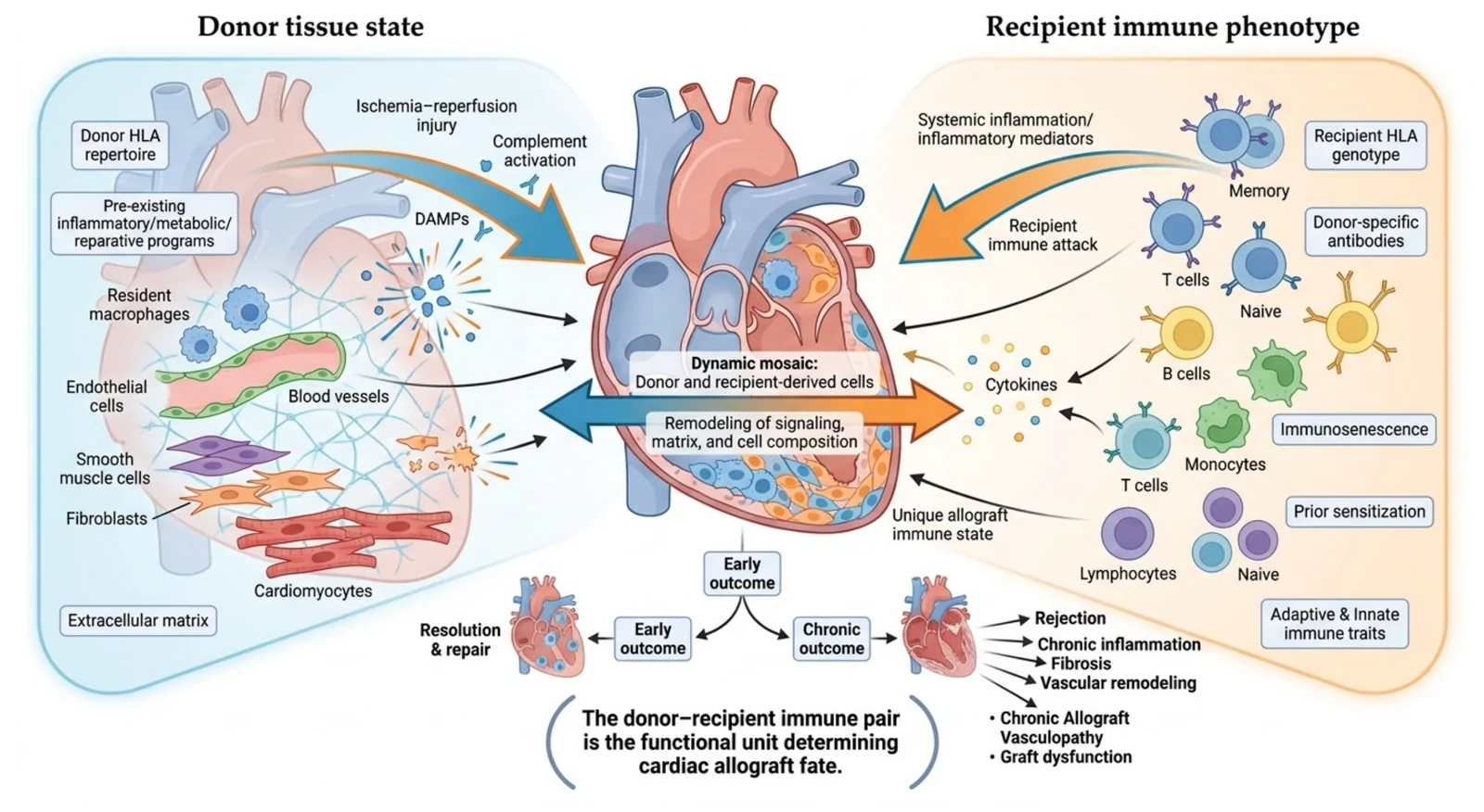
Interaction Between Donor Tissue State and Recipient Immunity After Heart Transplantation. Arrows: Black arrows directed toward the central allograft indicate the contribution of donor-derived tissue compartments and recipient-derived immune cells and mediators to the evolving post-transplant graft environment. The orange arrow on the donor side represents activation of the pre-existing donor tissue state by ischemia–reperfusion injury, DAMP release, and complement activation. The orange arrow on the recipient side represents recipient immune attack and the introduction of recipient-derived inflammatory and alloimmune signals into the graft. The large opposing blue and orange arrows within the central allograft indicate bidirectional donor–recipient interactions and progressive remodeling of cellular composition, intercellular signaling, and extracellular matrix, resulting in a dynamic mosaic of donor- and recipient-derived components. Arrows from the central allograft toward early outcomes represent divergence toward resolution and repair or persistence of injury. Arrows connecting early and chronic outcomes indicate that the early graft response may influence subsequent trajectories toward rejection, chronic inflammation, fibrosis, vascular remodeling, cardiac allograft vasculopathy, and graft dysfunction. Collectively, the arrows emphasize that allograft fate emerges from interaction between the pre-existing donor tissue state and the recipient immune phenotype rather than from either compartment in isolation. Abbreviations: DAMPs, damage-associated molecular patterns; HLA, human leukocyte antigen. Created by Urbanowicz T. with www.figurelabs.com, on 13 September 2026, certificate ID: FL-PUB-20260913-EOQE43.

The early allograft phenotype emerges from interactions among recipient immune cells and circulating mediators, donor-derived endothelium, graft-resident immune populations, and the myocardial tissue environment. Figure 3 summarizes this interaction, while Table 3 distinguishes established mechanisms from proposed donor–recipient interactions that remain to be validated clinically.

The biological consequences of transplantation may therefore depend less on isolated donor or recipient characteristics than on the context created by their combination. Features that are relatively well tolerated in one donor–recipient pairing may acquire greater significance in another, particularly when tissue susceptibility and recipient inflammatory potential converge. This interaction provides a useful framework for organizing variables currently assessed separately and for identifying those that may eventually prove informative at the donor–recipient pair level. The principal characteristics on each side of this interaction, along with their potential consequences for the emerging allograft phenotype, are summarized in Table 4.

Semidirect allorecognition provides a well-established example of such biological integration, because recipient antigen-presenting cells can display intact donor-derived MHC and thereby combine recipient cellular machinery with donor molecular determinants within a single alloimmune event [83,119].

A comparable integration occurs within the myocardial niche. Recipient monocytes entering the graft encounter graft-derived chemokines, extracellular matrix, endothelial signals, cellular debris, and resident stromal populations [120]. Their subsequent differentiation is influenced by interactions with graft fibroblasts and endothelial cells as well as other recipient-derived immune populations [121]. The post-transplant myocardial niche may therefore contain macrophage and stromal states that were present in neither the donor organ nor the recipient circulation before transplantation.

These interactions may also provide a mechanistic link between early inflammation and later graft remodeling. Initial donor–recipient interactions influence leukocyte recruitment, endothelial activation, and tissue repair, thereby altering the environment in which subsequent immune responses occur [122]. Recurrent injury may further modify macrophage–stromal and endothelial–immune interactions, favoring either resolution or persistent inflammation, fibrosis, and vascular remodeling [123]. This possibility provides a rationale for examining early immune states in relation to subsequent CAV rather than treating acute and chronic graft injury as entirely separate processes.

It would nevertheless be premature to conclude that donor immune memory is an established determinant of recipient alloimmunity in humans. Existing studies demonstrate that donor-resident immune cells are functionally relevant, that donor and recipient macrophage populations coexist after transplantation, and that recipient-derived cells acquire distinct phenotypes within the graft [121,124]. They have not yet established that a specific pre-donation exposure creates a durable donor immune program that subsequently alters recipient alloreactivity. This distinction is essential. The proposed model lies one mechanistic step beyond the current evidence.

Donor tissue obtained at procurement could be characterized using single-cell transcriptomic, epigenetic, and spatial methods and linked to recipient immune phenotyping prior to transplantation [125]. Cell-of-origin analysis after implantation could then determine how donor-resident populations disappear, persist, or influence the differentiation of incoming recipient cells [126]. Longitudinal integration with rejection phenotypes, donor-specific antibodies, molecular biopsy findings, fibrosis, and CAV would allow the contribution of donor state to be separated from established recipient risk factors [127].

Such studies would also test whether donor and recipient immune characteristics interact statistically and biologically rather than acting as independent predictors. This is an important distinction. Demonstrating that a donor macrophage phenotype predicts rejection would be informative; demonstrating that its effect depends on the recipient myeloid or adaptive immune phenotype would support a fundamentally different model of transplant risk.

The resulting framework can be expressed simply, but should not be interpreted simplistically: graft outcome reflects the interaction between donor tissue state, recipient immune state and the injuries imposed by transplantation and subsequent clinical events. HLA disparity determines an essential component of this relationship, but it does not capture its full biological context. The implication is that compatibility in heart transplantation may eventually require a broader definition. The future question may not be limited to whether a donor and recipient are immunologically compatible at the level of HLA and circulating antibodies, but whether particular donor tissue states are more or less permissive when paired with particular recipient immune phenotypes. This does not yet constitute a basis for organ allocation. It does, however, define a testable direction for precision transplant immunology.

In this model, transplantation is not the beginning of the graft’s immunological history. It is the point at which two histories converge. What follows—resolution, rejection, fibrosis or progressive vascular injury—may depend partly on how those histories interact within the newly formed allograft.

## 5. From Early Immune Activation to Chronic Allograft Injury

**Figure 4:**
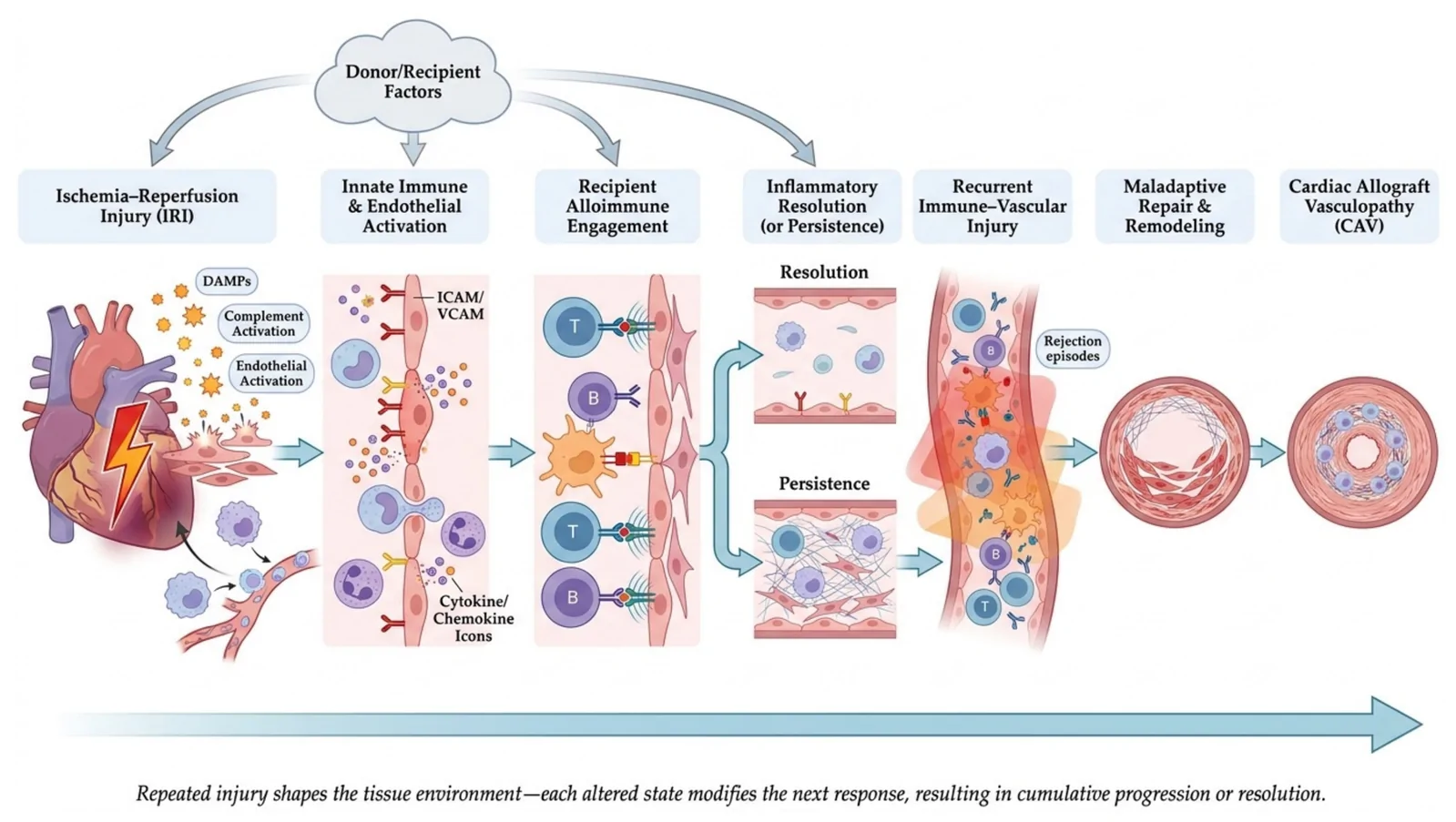
From Ischemia–Reperfusion Injury to Cardiac Allograft Vasculopathy: A Continuum of Immune and Vascular Remodeling. Arrows: The upper curved arrows originating from “Donor/Recipient Factors” indicate that pre-existing donor and recipient characteristics can influence multiple stages of the post-transplant response rather than acting at a single time point. The horizontal blue arrows indicate the proposed temporal sequence from ischemia–reperfusion injury through innate immune and endothelial activation, recipient alloimmune engagement, recurrent immune–vascular injury, maladaptive repair, and ultimately cardiac allograft vasculopathy. The branching arrows at the inflammatory-resolution stage indicate two alternative trajectories: effective resolution with restoration of tissue homeostasis, or persistence of inflammation with continued immune–stromal and extracellular-matrix remodeling. The arrows entering the recurrent immune–vascular injury compartment emphasize that persistent inflammation and repeated alloimmune episodes can converge on the graft vasculature. Subsequent arrows toward vascular remodeling and CAV represent cumulative structural consequences of recurrent endothelial and immune injury, including progressive intimal remodeling and luminal narrowing. The long arrow at the bottom represents the temporal continuum and cumulative nature of graft remodeling, emphasizing that each episode of injury may modify the tissue environment in which subsequent immune responses occur. Abbreviations: B, B lymphocyte; CAV, cardiac allograft vasculopathy; DAMPs, damage-associated molecular patterns; ICAM, intercellular adhesion molecule; IRI, ischemia–reperfusion injury; T, T lymphocyte; VCAM, vascular cell adhesion molecule. Urbanowicz T. with www.figurelabs.com, on 13 September 2026, certificate ID: FL-PUB-20260913-IQ8SHU.

The clinical distinction between early graft injury, acute rejection, and cardiac allograft vasculopathy (CAV) does not imply biological independence. Innate immune activation begins during reperfusion, whereas subsequent cellular and antibody-mediated responses repeatedly engage the graft vasculature and interstitium [128]. The outcome of each episode depends not only on the magnitude of injury but also on the effectiveness of inflammatory resolution and tissue repair. Recurrent or incompletely resolved injury can therefore alter the cellular composition and extracellular matrix of the graft, creating conditions that influence subsequent immune responses [6,129]. Viewed in this way, CAV represents the late vascular manifestation of cumulative and interacting immune, endothelial, and reparative processes rather than an isolated complication arising years after transplantation (Figure 4).

Ischemia–reperfusion is relevant to the present framework primarily because it establishes the inflammatory context in which alloantigen recognition begins. DAMP-dependent pattern-recognition pathways, complement activation, and graft-resident myeloid cells promote recruitment and activation of recipient leukocytes during the earliest phase after implantation [81]. Experimental transplantation studies further indicate that innate pathways activated during this period can influence subsequent alloimmune responses and chronic vascular pathology [130]. Early sterile inflammation should therefore be considered an immunological modifier of the graft rather than only a mechanism of acute tissue injury.

Early inflammation also modifies how donor antigens are encountered. Endothelial activation promotes leukocyte adhesion and transmigration, inflammatory mediators alter antigen-presenting cell function, and complement influences both the vascular and immune compartments [39]. Recipient myeloid cells therefore encounter donor antigens within a tissue already undergoing innate immune activation [131]. This provides a direct mechanistic link between sterile graft injury and the subsequent development of adaptive alloimmunity [132].

The endothelium is central to this progression because it is repeatedly exposed to recipient immune effectors throughout the graft’s lifespan. Following implantation, graft endothelial cells encounter complement, inflammatory cytokines, recipient leukocytes, and, when present, donor-specific antibodies (DSAs) [133]. During antibody-mediated rejection, endothelial cells are both targets of antibody binding and active participants in leukocyte recruitment and microvascular inflammation [134]. Repeated endothelial immune injury may therefore provide one route through which episodic alloimmunity is converted into persistent vascular remodeling [102].

CAV is often compared with accelerated atherosclerosis, but its pathology reflects a distinct transplant-specific environment [135]. Diffuse concentric intimal expansion develops within graft vessels that are repeatedly exposed to cellular alloimmunity, DSA (donor-specific antibody), complement activation, and non-immune cardiovascular risk factors [136]. Endothelial activation, macrophage and lymphocyte infiltration, vascular smooth muscle cell phenotypic modulation, and extracellular matrix deposition contribute to progressive luminal narrowing [102]. CAV is therefore not adequately classified as either an immune or a vascular disorder; it is a chronic vascular consequence of sustained interaction between the graft and recipient.

Experimental studies of CAV have demonstrated the expression of adhesion molecules and mismatched major histocompatibility complex (MHC) antigens on endothelial and medial vascular cells, in association with recipient macrophage and T-cell infiltration [137,138]. Human and experimental data further indicate that donor-derived vessel-wall cells and recipient-derived populations both contribute to the evolving neointimal lesion [139]. The vascular pathology of CAV can therefore be viewed as a tissue-level product of sustained interaction between graft-derived vascular structures and recipient immunity.

Macrophages provide an additional link between acute immune injury and chronic remodeling [140]. Their functions extend beyond inflammatory amplification to include the clearance of damaged cells, the regulation of leukocyte recruitment, antigen presentation, the resolution of inflammation, and the control of fibroblast activity and matrix remodeling [141,142]. Importantly, the macrophage compartment itself changes after transplantation as donor-resident populations decline and recipient-derived populations acquire graft-associated phenotypes. Persistent or maladaptive macrophage–stromal signaling may therefore contribute to the transition from recurrent immune injury to fibrosis and vascular remodeling [123].

The capacity to resolve inflammation may be as important as the intensity of its initiation. Successful resolution requires removal of damaged cells, attenuation of leukocyte recruitment, restoration of endothelial homeostasis, and coordinated repair of the extracellular matrix [143,144]. Failure of these processes allows inflammatory and reparative programs to coexist, promoting persistent macrophage activation, fibroblast engagement, and vascular remodeling [30,145]. In this setting, fibrosis is not simply the residue of previous injury but part of an evolving tissue environment that can influence subsequent immune responses [120,134].

In a cardiac allograft model, impairment of macrophage efferocytosis promoted CAV, whereas signaling through the efferocytosis receptor (MER Proto-Oncogene Tyrosine Kinase) MerTK suppressed vascular disease [146]. These observations are conceptually important because they move macrophage involvement beyond inflammatory burden alone. Efficient removal of apoptotic cells is part of tissue resolution; failure of this process allows cellular debris and inflammatory signaling to persist, thereby altering subsequent repair [147,148]. Chronic vascular injury may therefore reflect, at least partly, failure to terminate an inflammatory response appropriately rather than simply excessive initiation of that response.

The balance between injury and resolution may be one of the missing links between early graft inflammation and later structural disease. A vigorous innate response can be compatible with recovery if damaged cells are cleared, endothelial integrity is restored, and stromal activation subsides. The same initial insult may have very different consequences when apoptotic material persists, endothelial dysfunction remains, or macrophage–fibroblast signaling continues after the acute trigger has disappeared. Chronicity, in this model, is not determined by inflammation alone. It emerges when inflammatory and reparative programs fail to return the tissue to homeostasis.

Cytokines and growth factors generated during immune injury alter stromal-cell behavior, while endothelial dysfunction and immune-cell infiltration promote migration and proliferation of smooth-muscle-like cells within the intima [102,125]. Extracellular matrix accumulation gradually converts a predominantly inflammatory process into structural vascular disease [149]. As this occurs, the histological appearance of the lesion may become progressively less inflammatory even though immune injury was instrumental in its development [150]. This helps explain an apparent paradox of chronic transplantation pathology: the final fibrotic lesion may contain relatively little evidence of the biological events that initiated it.

Repeated subclinical or clinically recognized episodes of immune injury can influence interstitial fibrosis and myocardial remodeling even when ventricular function remains initially preserved [151,152]. The allograft thus accumulates the consequences of successive insults. Some are donor-derived, some recipient-derived, and others arise from transplantation itself. Their effects are unlikely to be simply additive. An early event may alter the tissue response to a later one.

This temporal dependence is central to the concept of immune memory. If a previous injury modifies macrophage, endothelial, or stromal responsiveness, then each subsequent insult occurs in tissue already altered by preceding events [16,121]. Ischemia–reperfusion, rejection, infection, metabolic stress, and therapeutic immunosuppression progressively reshape the graft, generating new cellular states that influence subsequent responses [18].

The resulting model differs from a linear sequence in which ischemia–reperfusion is followed by rejection and eventually by CAV. A more plausible biological architecture is iterative. Injury changes the tissue; the changed tissue modifies the next immune response; that response generates further injury and repair; and the resulting remodeling determines the context for the next event. With time, repeated iterations may stabilize as either relative tissue homeostasis or chronic inflammatory and fibrotic remodeling.

Such a model may also explain why conventional clinical variables predict late graft outcome only incompletely. Two recipients may have similar HLA mismatches, comparable immunosuppression, and apparently similar episodes of rejection, yet develop markedly different degrees of CAV. Conversely, substantial early inflammatory injury does not invariably lead to severe chronic disease. The difference may reside partly in properties that are not captured by the frequency of immune events: endothelial resilience, efficiency of inflammatory resolution, macrophage phenotype, stromal responsiveness, and the cumulative biological state of the graft.

There is strong evidence that ischemia–reperfusion activates innate pathways relevant to rejection; that endothelial injury is central to CAV; that donor and recipient cells contribute to vascular remodeling; and that macrophage function can influence experimental CAV. What has not yet been demonstrated is that a measurable donor tissue-memory state independently predicts the transition from early injury to chronic human allograft pathology. Establishing that link would require longitudinal studies beginning before implantation rather than analyses confined to the recipient after transplantation.

These considerations support a longitudinal view of allograft injury in which the biological significance of an early event is determined not only by its immediate severity, but also by the tissue context in which it occurs and by the quality of subsequent resolution and repair. Within this framework, procurement and reperfusion, alloimmune activation, endothelial dysfunction, and tissue remodeling represent interconnected stages rather than discrete complications. Their relative contribution is likely to change over time as donor-resident populations decline, recipient-derived cells accumulate, and the vascular and stromal compartments integrate successive inflammatory and metabolic insults. Table 5 summarizes this proposed continuum, highlighting the transition from the pre-existing donor tissue state through early post-transplant injury to the chronic vascular and fibrotic phenotype of the mature allograft.

CAV provides a particularly compelling model because its pathology preserves the history of this interaction [159,160]. The mature lesion contains donor vessel wall cells, recipient immune influences, extracellular matrix generated during repair, and the cumulative effects of immunological and metabolic injury [161]. By the time luminal narrowing is clinically apparent, these processes have become inseparable [162]. The lesion is neither exclusively donor-derived nor recipient-induced. It is the anatomical record of their interaction.

Seen from this perspective, the transition from early inflammation to chronic allograft disease is not simply a progression with time. Each episode of injury occurs within a tissue whose response has been conditioned by what preceded it. The donor heart enters this process with its first biological history already written; transplantation does not erase that history, but places it within a new immune environment in which it may be modified, amplified or eventually superseded.

## 6. Testing Donor Tissue Immune States in Human Heart Transplantation

The central experimental question arising from this review is whether a tissue immune state present before implantation can be distinguished from the changes induced by transplantation and subsequently shown to influence recipient immunity. Addressing this question requires temporal and cellular resolution. A molecular feature detected in the donor organ must first be shown to persist beyond the exposure associated with it and then be linked to an altered response after implantation. Studies limited to either the donor organ or the post-transplant allograft cannot establish this sequence.

Histologically similar endomyocardial biopsies can contain different molecular programs, and spatial transcriptomic analyses have identified distinct endothelial, stromal, and immune states within conventional rejection categories [22]. These approaches provide information that is not captured by morphology alone, but most studies begin only after implantation. At that point, the graft has already been exposed to reperfusion, recipient complement and leukocytes, immunosuppression, and potentially rejection or infection. A post-transplant molecular phenotype is therefore intrinsically composite and cannot, by itself, establish whether a particular state originated in the donor organ.

A pre-implantation myocardial sample provides the essential reference point for testing donor-state persistence. When linked to samples obtained after reperfusion and during subsequent surveillance, it allows molecular changes to be interpreted longitudinally rather than as isolated observations [163,164]. The purpose is not to derive another donor-risk score, but to determine whether reproducible donor-resident cellular states can be identified before implantation, whether they persist or leave measurable consequences after transplantation, and whether they modify the response to recipient immunity.

Ex vivo heart perfusion as an experimental window into donor tissue immune states. The increasing use of ex vivo heart perfusion (EVHP), particularly for assessing and preserving donation-after-circulatory-death (DCD) hearts and selected extended-criteria donor organs, provides a unique opportunity to investigate the donor tissue state before implantation [165]. In contrast to static preservation, normothermic perfusion provides a temporally defined, experimentally accessible interval during which the myocardium remains metabolically active and can be repeatedly sampled under controlled conditions. Serial analysis of perfusate and, where feasible, myocardial tissue could therefore determine whether inflammatory, metabolic, endothelial, or myeloid phenotypes present at procurement persist, resolve, or evolve during perfusion. Integration of EVHP with proteomic and metabolomic profiling, perfusate biomarker analysis, and targeted myocardial sampling could provide a dynamic rather than purely cross-sectional characterization of the donor organ [166]. Particularly informative would be paired sampling at procurement, during EVHP, immediately before implantation, and after reperfusion, thereby separating donor-intrinsic states from changes induced by preservation and recipient exposure. DCD hearts may be especially relevant to this framework because warm ischemia and subsequent reperfusion introduce a defined sequence of metabolic and inflammatory stresses that can be studied longitudinally [167]. EVHP should therefore be considered not only as a preservation and viability assessment platform but also as a potential experimental bridge between donor biology and the post-transplant immune phenotype.

Beyond phenotyping, EVHP may eventually provide an opportunity for controlled pre-implant intervention. Because treatment can theoretically be directed to the isolated donor organ without exposing the recipient systemically, the perfusion interval offers a potential platform for modifying inflammatory and metabolic pathways before implantation [162]. Candidate strategies could include removal or neutralization of soluble inflammatory mediators, modulation of macrophage or endothelial activation, optimization of mitochondrial and redox metabolism, and targeted delivery of pharmacological or biological agents [168]. Within the framework proposed here, such interventions would provide an especially informative experimental test: if a defined donor-tissue state can be identified during perfusion, selectively modified, and subsequently linked to a different response after reperfusion, this would provide stronger evidence of functional relevance than association alone. At present, however, immune reprogramming during EVHP should be regarded as an experimental direction rather than an established therapeutic strategy.

Ex vivo normothermic heart perfusion provides a particularly attractive platform for testing this framework by creating a temporally defined interval in which the donor organ can be studied independently of the recipient [169,170]. Serial perfusate and myocardial sampling could track inflammatory, metabolic, and transcriptional trajectories from procurement to implantation and, when integrated with single-cell, spatial, proteomic, and metabolomic profiling, determine which donor-tissue states persist during perfusion [171,172]. Beyond observation, EVHP offers a controlled experimental window in which candidate states could potentially be modified before recipient exposure, for example through metabolic conditioning or targeted modulation of inflammatory pathways. Whether such interventions can safely alter tissue-resident programs and subsequently influence recipient alloimmunity remains to be established prospectively. This opportunity may be particularly relevant to DCD transplantation, in which warm ischemia, reperfusion, and machine-perfusion exposure create a distinct sequence of metabolic and inflammatory stresses before implantation. Comparative longitudinal profiling of DCD and DBD hearts during ex vivo perfusion could determine whether procurement and preservation pathways modify the donor-tissue state ultimately encountered by the recipient.

Single-cell and single-nucleus approaches are particularly suited to this question because the relevant signal is unlikely to be uniform across the myocardium [93,121]. Bulk transcriptional profiling cannot reliably distinguish activation of resident macrophages from changes in leukocyte abundance, endothelial activation, or stromal remodeling [112]. Cell-resolved analysis can separately define these compartments and, when combined with donor–recipient genotyping, assign cellular provenance after transplantation [16]. This distinction is essential: the persistence of a donor-derived program, the acquisition of a new phenotype by recipient cells, and the convergence of both processes represent biologically distinct mechanisms.

Experimental manipulation of donor-resident macrophages provides proof of principle that a cellular state present in the graft before implantation can alter subsequent recipient alloimmunity [88]. These studies justify prospective investigation of donor-resident immune populations, but they do not establish that such states reflect memory of an antecedent donor exposure.

Transcriptional state alone is insufficient to establish immune memory because gene expression can change rapidly during procurement and reperfusion [173]. Evidence for persistent programming would be strengthened by regulatory features that remain detectable after the initiating exposure has resolved [174]. Chromatin accessibility, histone modifications, and DNA methylation are therefore particularly relevant when interpreted together with transcriptional and functional data [175]. The critical distinction is between acute activation and a durable regulatory state that alters the response to a later challenge.

The vascular compartment offers a complementary opportunity to test donor-state persistence because graft endothelium provides a relatively durable interface with recipient immunity [176]. Molecular studies of antibody-mediated rejection have identified endothelial, NK-cell, and inflammatory transcriptional programs associated with DSA and microvascular injury [30]. A longitudinal design could extend these observations by asking whether endothelial states detectable before implantation are associated with the character or persistence of subsequent endothelial alloimmune responses [172].

Human CAV provides an example of the information gained by cell-resolved and spatial analysis [19]. Single-cell and spatial studies have identified modulated vascular smooth-muscle cells, macrophage populations, type I interferon signaling, and regionally distinct inflammatory and remodeling programs within diseased coronary arteries. These findings demonstrate that the mature CAV lesion contains organized cellular interactions rather than a homogeneous vascular phenotype. Longitudinal studies are now required to determine when these states emerge and whether any can be traced to cellular or molecular features present before implantation.

Spatial information is necessary because myocardial immune and stromal states are anatomically heterogeneous. Perivascular macrophages, capillary endothelial cells, interstitial fibroblasts, and infiltrating immune populations occupy distinct local niches. Dissociation-based methods resolve cellular identity but remove much of this anatomical context. Spatial transcriptomic or proteomic analysis can therefore determine whether candidate persistent states are restricted to particular vascular, perivascular, or fibrotic regions and whether these regions subsequently become sites of recurrent inflammation or remodeling [101,177].

Donor and recipient cells coexist after transplantation, and their relative contribution changes over time [178]. Genetic variants, sex-chromosome markers in sex-mismatched transplants, and other lineage-informative approaches can be integrated with single-cell data to distinguish donor- from recipient-derived populations [179]. This is essential for determining whether a post-transplant state represents persistence of donor biology, a phenotype newly acquired by recipient cells, or convergence of both. For macrophages in particular, cellular replacement should be studied together with phenotypic transition: the relevant question is not only which cells remain donor-derived, but how recipient-derived cells differentiate after entering the graft.

Longitudinal spatial analysis of rejection also suggests that the clinically relevant phenotype may extend beyond the presence or absence of rejection. Distinct cellular and signaling programs have been observed in biopsies from episodes that subsequently resolve compared with those that persist. Demonstrating such an association, particularly if linked to a persistent donor-derived program, would provide substantially stronger evidence for functional donor tissue conditioning than a cross-sectional inflammatory signature alone.

Circulating and tissue-based surveillance assays provide complementary longitudinal information. dd-cfDNA is sensitive to graft injury but does not identify its cellular mechanism, whereas DSA, gene-expression profiling, molecular biopsy analysis, and histology interrogate different components of alloimmune activity and tissue injury [180]. Their value for the present hypothesis lies in temporal anchoring: they can define when graft injury or immune activation emerges relative to a pre-implantation tissue state. None can establish donor-state persistence in isolation.

Longitudinal spatial analysis of rejection provides early evidence that such tissue states are clinically meaningful. In human heart-transplant biopsies, spatial transcriptomics has demonstrated substantial molecular heterogeneity within conventional histological rejection categories and distinct cellular programs between patients who subsequently respond to augmented immunomodulatory therapy and those with persistent rejection [6]. In this analysis, pathways involving IL-6–JAK–STAT3, type I and type II interferon responses, TNF signaling and several cellular compartments differed according to subsequent rejection behavior. The clinically relevant phenotype may therefore not be simply the presence of rejection, but the tissue’s capacity either to resolve or to sustain it.

Circulating biomarkers provide a complementary, but different, level of information. Donor-derived cell-free DNA has emerged as one of the most useful non-invasive indicators of cardiac allograft injury. In a prospective cohort of 223 adult recipients, dd-cfDNA was substantially higher during acute rejection and, at the selected threshold, provided a negative predictive value of 97.3% [181]. A subsequent multicentre prospective study similarly found a high negative predictive value for acute cellular rejection, while also demonstrating the important limitation that an elevated value does not identify the biological cause of graft injury with equivalent precision.

A rigorous test of the donor-state hypothesis requires three linked datasets: a pre-implantation graft profile, a recipient immune profile, and longitudinal characterization of the same allograft after implantation. The first defines the candidate donor tissue state; the second defines the immune environment into which the graft is introduced; and the third determines whether the resulting tissue trajectory is associated with either component or, importantly, with their interaction. The analytical objective is therefore not maximal molecular profiling, but attribution: which features were already present in the donor, which arose in recipient-derived cells, and which emerged only after the two compartments interacted?

**Figure 5:**
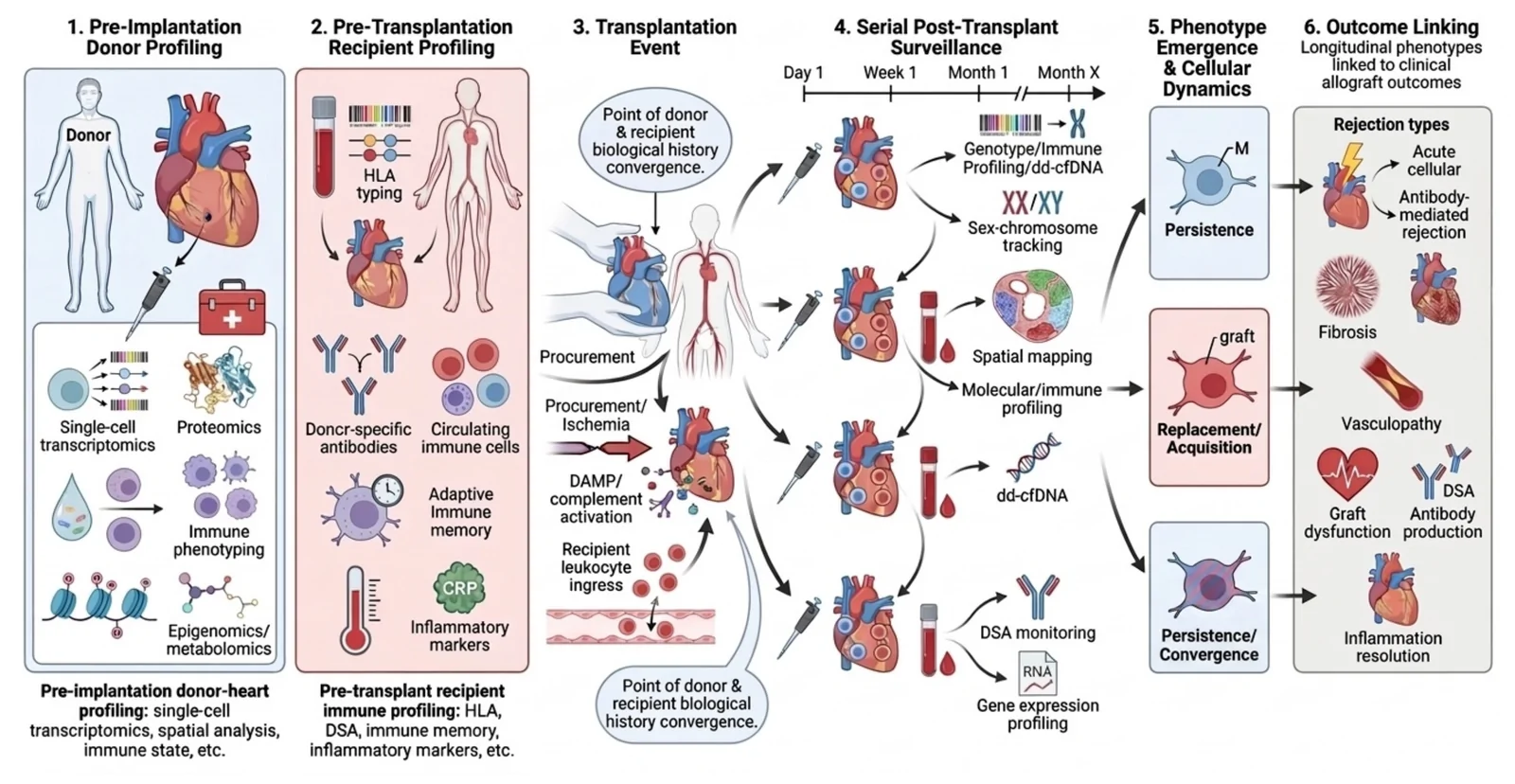
Longitudinal Reconstruction of Donor–Recipient Immune Interactions After Heart Transplantation. Arrows: The left-to-right arrows across the six panels indicate the proposed longitudinal study sequence, beginning with paired donor and recipient profiling before transplantation and extending through the transplantation event, serial post-transplant surveillance, assessment of cellular persistence or replacement, and linkage to clinically relevant allograft phenotypes. Arrows converging at the transplantation event indicate the point at which the independently acquired biological histories of the donor tissue and recipient immune system first interact. Arrows from transplantation to serially sampled allografts and blood specimens represent longitudinal acquisition of tissue and circulating biomarkers at predefined post-transplant time points. Branching arrows within the surveillance panel indicate complementary approaches for resolving cellular origin, spatial localization, molecular phenotype, and ongoing alloimmune activity. Arrows from serial surveillance to phenotype emergence indicate longitudinal assessment of whether pre-existing donor states persist, are replaced or acquired by recipient-derived cells, or converge toward shared graft phenotypes. Final arrows toward clinical outcomes indicate linkage of these cellular and molecular trajectories to rejection, fibrosis, vasculopathy, graft dysfunction, antibody production, and inflammatory resolution. Collectively, the arrows define an experimental strategy for distinguishing true persistence of donor-derived biological states from post-transplant cellular replacement and newly acquired recipient-driven changes. Abbreviations: CRP, C-reactive protein; dd-cfDNA, donor-derived cell-free DNA; DSA, donor-specific antibodies; HLA, human leukocyte antigen; M, macrophage; RNA, ribonucleic acid; X/Y, X- and Y-chromosome markers. Created by Urbanowicz T. with www.figurelabs.com, on 13 September 2026, certificate ID: FL-PUB-20260913-G7ZY56.

A clinically meaningful model of precision transplantation will require these dimensions to be considered together. The pre-implant state of the donor heart provides the biological starting point, recipient immunity determines the context in which the graft is subsequently challenged, and longitudinal surveillance records the phenotype that emerges from their interaction. Linking these observations across time may allow graft injury to be interpreted as a biological trajectory rather than as a succession of isolated post-transplant events (Figure 5).

The first would require tissue-level characterization of the donor heart [182]. The second would incorporate established alloimmune variables—HLA mismatch, DSA and sensitization—but could ultimately extend to recipient immune-cell composition, inflammatory phenotype, hematopoietic clonal architecture and previous immune exposure. The third would use serial measures of tissue injury and immune activity, including dd-cfDNA, circulating immune signatures, molecular biopsy phenotypes and, where appropriate, spatial or single-cell analysis.

Translating this framework into a testable clinical model will require complementary measurements obtained at biologically informative time points rather than reliance on a single molecular platform. The principal challenge is to distinguish features already present in the donor organ from those introduced by the recipient or acquired during the post-transplant course, while retaining information on cellular origin, tissue organization, and temporal change. A multimodal strategy may therefore be most informative when each method addresses a defined component of this trajectory and the resulting data are interpreted in relation to conventional clinical and immunological variables. The principal approaches that could contribute to such longitudinal characterization of the donor–recipient allograft system are summarized in Table 6.

The immediate value of this framework is experimental rather than allocative. A candidate donor immunotype should first demonstrate reproducibility, biological persistence, and an association with post-transplant phenotype that is independent of established donor and recipient risk factors. More importantly, studies should test interaction terms explicitly [187,188]. A donor tissue state that has little prognostic value across an entire cohort may nevertheless be biologically relevant if its effect depends on a defined recipient immune phenotype. Prospective studies will therefore require paired donor–recipient analysis rather than conventional models in which donor and recipient variables are treated only as independent covariates [189].

The demonstration that donor CCR2-positive macrophages can facilitate recipient alloreactive responses through MYD88-dependent signaling identifies a donor-resident pathway that is biologically upstream of conventional rejection [45]. The identification of macrophage–vascular smooth-muscle interactions and type I interferon signaling within human CAV similarly provides a tissue-level mechanism rather than a descriptive biomarker [18]. These observations point toward a different form of transplant precision medicine: one based not simply on predicting rejection, but on identifying which biological circuit is dominant in a particular graft.

Ex vivo perfusion may provide the most direct translational setting in which this framework can eventually be tested. In DCD and other donor hearts undergoing EVHP, the interval between procurement and implantation creates an unusual opportunity to characterize the organ repeatedly while it remains outside the recipient [190]. This could enable donor-tissue phenotyping to shift from a static baseline measurement to the assessment of dynamic biological responsiveness [191]. More importantly, EVHP could ultimately provide a platform for identifying and experimentally modifying a candidate’s adverse tissue state before implantation. The progression from phenotyping → functional challenge → targeted intervention → post-transplant follow-up would provide a particularly rigorous strategy for determining whether donor tissue states are merely biomarkers of previous injury or modifiable determinants of subsequent allograft biology.

## 7. Conclusions and Future Perspectives

Heart transplantation creates a biological system in which donor-derived tissue and recipient immunity coexist and progressively reshape one another. The graft is not simply a source of alloantigen. Resident macrophages, vascular and stromal cells, extracellular matrix, and other components of the myocardial niche accompany the organ into the recipient and participate in the earliest responses to transplantation. Recipient-derived immune cells subsequently enter this environment, replace parts of the donor immune compartment, and acquire phenotypes influenced by conditions within the graft. The post-transplant immune state therefore emerges from interactions between cellular populations of different origin rather than from either donor or recipient biology alone.

Available evidence supports several elements of this model. Donor-resident macrophage populations can influence recipient leukocyte recruitment and alloimmune activation; donor and recipient macrophages coexist during the evolution of the cardiac allograft; and recipient-derived macrophages do not necessarily reproduce the phenotype of the donor-resident populations they replace. The vascular and stromal compartments provide additional sites at which recipient immune responses are translated into tissue injury, repair, fibrosis, and vascular remodeling. Human single-cell and spatial studies of rejection and CAV increasingly demonstrate that these processes are organized within discrete cellular and anatomical niches rather than occurring uniformly throughout the graft.

A more speculative question is whether the donor heart carries persistent consequences of exposures that occurred before transplantation. Evidence of inflammation at procurement, associations between donor characteristics and outcome, or differences in baseline gene expression are insufficient to establish such memory. Demonstration of donor tissue immune memory would require a defined antecedent exposure, persistence of a molecular or functional alteration after that exposure has resolved, and evidence that the persistent state modifies the response to a subsequent challenge. These criteria have not yet been satisfied in human heart transplantation. The concept should therefore remain a testable hypothesis rather than an assumed property of donor organs.

Importantly, persistence need not require indefinite survival of the original donor immune cells. A transient donor-resident population could alter endothelial activation, stromal behavior, extracellular-matrix organization, or the recruitment and differentiation of recipient cells before disappearing. In this setting, biological continuity would reside partly within the tissue niche and the sequence of cellular interactions rather than within a permanently retained cell population. This possibility provides a mechanistic basis for studying cellular succession alongside conventional measures of cellular persistence.

Testing this hypothesis will require prospective studies that begin before implantation. Pre-implant donor tissue should be linked to early reperfusion samples, determination of donor or recipient cellular origin, serial blood and tissue surveillance, rejection phenotype, and long-term vascular outcomes. Single-cell and spatial methods are particularly well suited to this purpose when they are used to answer defined questions of cellular provenance, persistence, and tissue organization rather than simply to generate increasingly detailed molecular descriptions. The decisive analysis will be longitudinal: whether a state identified in donor tissue remains biologically traceable after transplantation and whether its consequences can be distinguished from those produced by ischemia–reperfusion, recipient immunity, immunosuppression, and subsequent graft injury.

This approach also changes how donor and recipient factors should be investigated experimentally. Their effects should not always be assumed to be independent. A donor tissue state may become consequential only in the presence of a particular recipient immune phenotype, while the same state may have little measurable effect in another immunological context. Future studies should therefore examine donor × recipient interactions explicitly and determine whether they explain variation in rejection resolution, chronic inflammation, fibrosis, or CAV beyond established clinical and immunological risk factors. At present, this concept has no validated role in donor allocation or recipient matching.

## Figures and Tables

**Table 1:** Cellular Components of the Donor Heart as an Immunologically Active Organ.

Cellular Compartment	Principal Physiological Role in the Heart	Potential Immunological Function in the Donor Graft	Potential Relevance After Transplantation	References
Tissue-resident macrophages	Tissue surveillance, clearance of cellular debris, maintenance of tissue integrity, extracellular matrix turnover, vascular homeostasis, and repair	Sense tissue injury and danger signals; regulate local inflammatory responses; interact with endothelial and stromal cells; may influence recruitment and activation of recipient-derived immune cells	Donor-resident macrophages are present at reperfusion and may influence the earliest response to ischemia–reperfusion injury and subsequent alloimmune activation; their progressive replacement by recipient-derived macrophages may reshape the graft immune niche	[26,27]
Dendritic cells and other resident antigen-presenting cells	Immune surveillance and maintenance of local immune homeostasis	Capture and process antigens and participate in communication between innate and adaptive immunity	May contribute to early recognition of donor antigens and initiation or amplification of recipient adaptive immune responses	
Tissue-resident lymphocytes	Local immune surveillance and rapid tissue-level immune responses	Provide locally positioned adaptive immune activity and may retain phenotypic features associated with previous antigenic or inflammatory exposure	Could contribute to the initial inflammatory environment of the graft and represent a potential cellular substrate for persistence of donor-acquired immune states	[28,29]
Endothelial cells	Regulation of vascular integrity, permeability, vascular tone, thrombosis, and leukocyte trafficking	Sense hypoxia, oxidative stress, complement, cytokines, and metabolic injury; regulate leukocyte adhesion and transmigration; participate directly in alloimmune interactions	Form the persistent donor-derived interface with recipient blood and immunity; endothelial activation may connect ischemia–reperfusion injury with rejection and, over time, chronic vascular remodeling and CAV	[30,31]
Cardiac fibroblasts and stromal cells	Extracellular matrix maintenance, structural support, tissue repair, and remodeling	Respond to inflammatory and mechanical signals and produce cytokines, chemokines, growth factors, and matrix components; influence leukocyte recruitment and macrophage phenotype	May determine whether inflammation resolves or progresses toward persistent immune activation, extracellular matrix remodeling, and fibrosis	[9,32]
Cardiomyocytes	Contractile function and maintenance of myocardial performance	Respond to ischemic, metabolic, and inflammatory stress and release damage-associated and soluble signals into the local tissue environment	Tissue injury may amplify innate immune activation and modify the myocardial environment in which donor- and recipient-derived immune cells interact	[33,34]

Abbreviations: CAV, cardiac allograft vasculopathy.

**Table 2:** Operational Definitions Used in the Proposed Donor-Tissue Framework.

Term	Operational Definition	Persistence Required?	Secondary Response Required?	Interpretation	References
Baseline donor phenotype	Molecular, cellular, metabolic, or structural state present in the donor heart at procurement	No	No	Descriptive donor characteristic; does not establish memory	--
Donor-tissue conditioning	Modification of the graft state by antecedent biological exposures	Not necessarily	No	Exposure-related conditioning; may be transient or persistent	--
Persistent donor state	Exposure-associated cellular or tissue alteration remaining after the initiating stimulus has resolved	Yes	No	Demonstrates persistence, but not by itself immune memory	--
Trained innate immunity	Persistent epigenetic, transcriptional, and metabolic reprogramming of innate immune cells producing altered responsiveness to subsequent stimulation	Yes	Yes	Cell-intrinsic innate immune memory	[50,53,53,57,58]
Structural/matrisomal imprint	Persistent ECM, stromal, endothelial, or architectural alteration influencing subsequently arriving cells	Yes	No autonomous recall response	Tissue-level persistence; should not itself be termed immune memory	[63,64]
Donor-heart immune memory	Exposure-induced persistent cellular program in the donor heart that produces an altered response to a defined subsequent challenge	Yes	Yes	Highest evidentiary threshold proposed in this review	--

Note: The terms baseline donor phenotype, donor-tissue conditioning, persistent donor state, structural/matrisomal imprint, and donor-heart immune memory are used as operational categories within the conceptual framework proposed in this review. References are provided where definitions incorporate established biological concepts. Absence of a reference indicates a proposed operational definition rather than an independently established biological entity. Abbreviations: ECM, extracellular matrix.

**Table 3:** Candidate Mechanisms, Current Evidence Base, Interpretative Status, and Experimental Criteria for Persistent Donor-Tissue Immune States.

Candidate Mechanism	Potential Cardiac Compartment	What Must Persist	Subsequent Challenge	Evidence Needed to Establish Donor-Heart Immune Memory	Current Evidence Base	Established/ Supported Indirectly/ Hypothetical	References
Trained innate immunity	Resident macrophages/myeloid cells	Altered chromatin/transcriptional responsiveness after stimulus resolution	IRI, DAMPs, recipient cytokines	Exposure-linked persistent program plus altered secondary response	Non-transplant cardiac biology; extrapolative mechanistic evidence	Supported indirectly	[71,72]
Tissue-resident adaptive memory	Tissue-resident lymphocytes	Antigen-experienced resident population	Antigen/inflammatory restimulation	Persistence and functional recall response	Non-transplant cardiac biology; extrapolative mechanistic evidence	Supported	[73,74]
Endothelial inflammatory memory	Endothelium	Stable alteration in inflammatory responsiveness	Complement, DSA, cytokines	Pre-existing state predicting altered post-transplant response	Non-transplant cardiac biology; extrapolative mechanistic evidence	indirectly	[75,76]
Persistent stromal/matrisomal imprint	Fibroblasts/ECM	Persistent stromal or matrix state	Recipient-cell infiltration/inflammation	Demonstrable effect on recipient-cell behavior	Non-transplant cardiac biology; extrapolative mechanistic evidence	Supported	[77,78]
Persistent multicellular niche remodeling	Immune–vascular–stromal network	Stable intercellular program	Reperfusion/recipient immunity	Spatially conserved program linked to subsequent graft phenotype	Human heart transplantation; experimental cardiac transplantation; extrapolative mechanistic evidence	indirectly	[79,80]
Donor-resident macrophage functional state	Donor-derived cardiac macrophages	An exposure-induced macrophage program persisting after resolution of the antecedent stimulus	IRI, DAMPs, recipient-cell infiltration/alloimmune activation	Demonstration that the functionally relevant donor macrophage phenotype derives from a defined antecedent exposure, persists after its resolution, and alters the response to transplantation-associated challenge	Experimental cardiac transplantation; human heart transplantation	Established for functionality; hypothetical for donor-cell memory	[81,82]

Note: Current evidence base identifies the experimental or clinical setting from which supporting evidence is derived, whereas interpretative status indicates the extent to which that evidence directly supports the proposed mechanism in the donor heart. “Established” denotes direct evidence for the stated biological process; “supported indirectly” denotes mechanistically relevant evidence that does not directly establish the proposed process in the human donor heart; and “hypothetical” denotes a testable extension for which direct evidence is currently lacking. Abbreviations: DAMPs, damage-associated molecular patterns; DSA, donor-specific antibodies; ECM, extracellular matrix; IRI, ischemia–reperfusion injury.

**Table 4:** Donor and Recipient Immune Characteristics Potentially Shaping the Post-Transplant Allograft Phenotype.

Biological Domain	Donor-Side Determinant	Recipient-Side Determinant	Proposed Donor–Recipient Interaction	Potential Allograft Consequence	Evidence Status/Interpretation	References
Alloantigenic disparity and adaptive immunity	Donor HLA expression and antigenic repertoire	HLA mismatch, preformed or de novo DSA, previous sensitization, memory B- and T-cell responses	Donor alloantigens are encountered within a recipient immune system with a pre-existing degree of antigen-specific memory and humoral reactivity	Cellular or antibody-mediated alloimmune injury; endothelial activation; recurrent rejection	Established component of transplant immunology; the proposed framework extends rather than replaces conventional histocompatibility	[107,108]
Donor-resident innate immunity	Composition and activation state of resident macrophages and other innate immune populations	Recipient monocyte/macrophage inflammatory responsiveness and circulating myeloid phenotype	Donor-resident cells may influence early chemokine production, antigen-presenting cell recruitment, and the inflammatory environment in which recipient myeloid cells enter the graft	Differences in early innate activation, recipient-cell recruitment, inflammatory resolution, and subsequent alloimmune amplification	Supported by experimental transplantation studies; magnitude and clinical relevance in humans require prospective validation	[109,110]
Endothelial–immune interface	Endothelial activation, senescence, mitochondrial reserve, inflammatory responsiveness, and susceptibility to complement-mediated injury	DSA, complement activity, circulating leukocyte phenotype, systemic inflammatory state	Persistent donor endothelium determines the vascular context in which recipient antibodies and leukocytes encounter donor antigens and gain access to the graft	Endothelial dysfunction, leukocyte recruitment, microvascular injury, antibody-mediated injury, and potentially chronic vascular remodeling	Strong biological and clinical rationale; contribution of a pre-existing donor endothelial state remains incompletely defined	[31,111]
Ischemia–reperfusion susceptibility	Pre-existing myocardial, endothelial, mitochondrial, and resident immune-cell state	Recipient complement, neutrophil and monocyte responses; systemic inflammatory milieu	A comparable ischemic insult may generate different inflammatory responses depending on donor tissue resilience and recipient effector responsiveness	Variable severity of reperfusion injury and different inflammatory starting conditions for subsequent alloimmunity	Ischemia–reperfusion biology is established; donor × recipient interaction as a determinant of response remains a testable hypothesis	[112,113]
Innate and hematopoietic conditioning	Donor tissue conditioning related to age, metabolic stress, inflammation, or previous injury	Recipient age, chronic inflammation, metabolic disease, immunosenescence, and potentially clonal hematopoietic architecture	Recipient cells with different inflammatory thresholds enter donor tissues that themselves differ in basal activation and reparative capacity	Divergent macrophage differentiation, cytokine signaling, inflammatory persistence, or resolution despite similar conventional immunological risk	Mechanistically plausible; direct evidence for clinically relevant interaction is limited	[114,115]
Stromal and extracellular-matrix signaling	Fibroblast phenotype, extracellular-matrix organization, and pre-existing stromal activation	Recipient macrophages, lymphocytes, and inflammatory mediators entering the myocardial niche	Donor-derived stromal signals may condition the recruitment, localization, and differentiation of recipient immune populations; recipient inflammation may reciprocally drive fibroblast activation	Resolution and repair versus persistent inflammation, extracellular-matrix deposition, and fibrosis	Supported by tissue-immunology principles; transplant-specific donor–recipient interactions remain insufficiently characterized	[100,116]
Semidirect allorecognition	Intact donor MHC molecules and donor-derived extracellular vesicles	Recipient antigen-presenting cells and directly alloreactive T cells	Recipient APCs acquire intact donor MHC and present it to recipient T cells, creating an immune interaction that incorporates material from both donor and recipient compartments	Amplification or maintenance of recipient alloreactive T-cell responses	Established mechanistic pathway illustrating direct biological integration of donor- and recipient-derived components	[117,118]
Formation of the post-transplant myocardial niche	Persistent donor endothelium and stromal architecture; transient or persistent donor-resident immune populations	Progressive influx and differentiation of recipient monocytes, macrophages, lymphocytes, and other circulating cells	Recipient-derived cells acquire tissue-adapted phenotypes within a donor-derived niche already modified by procurement, reperfusion, and early inflammation	Emergence of cellular states not present in either donor or recipient before transplantation; establishment of a new allograft immune ecosystem	Supported conceptually and by cellular studies; causal relationship with clinical outcomes requires longitudinal human data	[100,108],
Inflammatory resolution and tissue repair	Endothelial resilience, macrophage state, stromal responsiveness, and capacity for tissue repair	Magnitude and persistence of recipient cellular and humoral immune activation	The balance between donor tissue resilience and recipient inflammatory pressure may determine whether an immune episode resolves or becomes biologically self-sustaining	Recovery of tissue homeostasis versus persistent inflammation, fibrosis, and progressive vascular injury	Central mechanistic hypothesis of the proposed donor–recipient interaction model	[100,108],
Long-term vascular remodeling	Persistent donor-derived vascular cells and their acquired or pre-existing biological state	Recurrent cellular/humoral alloimmunity, systemic inflammation, metabolic exposure, and recipient-derived vascular inflammatory cells	Repeated interaction between donor vascular structures and recipient immune effectors progressively reshapes the vascular niche	Fibrointimal remodeling and CAV	Multifactorial relationship well established; specific contribution of donor immune memory remains unproven	[101,102]

Abbreviations: APC, antigen-presenting cell; CAV, cardiac allograft vasculopathy; DSA, donor-specific antibodies; HLA, human leukocyte antigen; MHC, major histocompatibility complex.

**Table 5:** From Early Graft Injury to Cardiac Allograft Vasculopathy: Proposed Mechanistic Continuum Linking Donor Tissue State to Chronic Allograft Remodeling.

Stage of the Continuum	Principal Biological Event	Key Cellular and Molecular Mechanisms	Potential Contribution of Donor Tissue State	Consequence for Subsequent Graft Trajectory	Evidence Status	Ref.
Pre-implantation tissue immune state	The donor heart enters transplantation with an established resident immune, vascular, and stromal compartment	Tissue-resident macrophage heterogeneity; endothelial activation state; stromal phenotype; extracellular-matrix organization; local inflammatory programming	Previous donor exposures may alter the functional state of resident macrophages, endothelial cells, and stromal populations before transplantation	Establishes heterogeneity in the tissue environment encountered by recipient immunity after implantation	Donor-resident immune heterogeneity is established; persistence of exposure-induced donor immune memory in human HTx remains unproven	[17]
Reperfusion-associated innate immune activation	Sterile tissue injury activates graft-resident and recruited innate immune pathways	DAMP release; TLR and inflammasome signaling; complement activation; donor-resident macrophage activation; neutrophil and monocyte recruitment	Pre-existing differences in graft-resident immune and endothelial states may modify the magnitude or composition of the early inflammatory response	Establishes the inflammatory context in which donor alloantigens are initially encountered	Strong experimental evidence for innate activation after reperfusion; contribution of pre-existing donor immune programming remains hypothetical	[82]
Endothelial activation and recipient leukocyte recruitment	Graft endothelium becomes an active interface between tissue injury and recipient immunity	Adhesion-molecule expression; chemokine signaling; complement; leukocyte tethering, adhesion, and transmigration; endothelial antigen presentation	Baseline endothelial inflammatory state may influence recruitment and activation of recipient leukocytes	Converts sterile graft injury into a cellular environment permissive to alloimmune activation	Strong human and experimental evidence for endothelial involvement in rejection and CAV	[153]
Donor–recipient innate immune interaction	Donor-resident myeloid cells transiently coexist and interact with infiltrating recipient-derived cells	Donor macrophage MYD88-dependent signaling; recipient monocyte recruitment; macrophage differentiation within the graft; cytokine and chemokine networks	Functional heterogeneity among donor-resident macrophages may influence the recruitment and activation of recipient immune populations	Shapes the early composition and functional state of the post-transplant immune niche	Direct experimental cardiac-transplant evidence; human data demonstrate donor–recipient macrophage coexistence and progressive cellular replacement	[140]
Adaptive alloimmune amplification	Recipient T- and B-cell responses become established within the graft	Direct, indirect, and semidirect allorecognition; APC–T-cell interactions; cytotoxic T-cell activity; B-cell activation and DSA formation	Donor-derived APCs, MHC-bearing material, endothelium, and local inflammatory signals influence the context of alloantigen presentation	Determines the magnitude, phenotype, and persistence of cellular and humoral alloimmunity	Mechanisms of allorecognition are well established; quantitative influence of donor tissue state remains incompletely defined	[154]
Endothelial alloimmune injury and AMR	Humoral and cellular immune effectors repeatedly engage the graft microvasculature	DSA binding; complement activation; endothelial signaling; NK-cell and macrophage recruitment; microvascular inflammation	The graft vascular compartment provides the donor-derived substrate on which recipient humoral immunity acts	Recurrent endothelial injury promotes microvascular dysfunction and creates conditions favoring chronic vascular remodeling	Strong clinical and molecular evidence linking DSA, endothelial injury, AMR, and adverse graft outcomes	[155,156]
Resolution versus persistence of inflammation	The graft either restores tissue homeostasis or retains an activated immune–stromal environment	Clearance of injured cells; attenuation of leukocyte recruitment; macrophage state transitions; endothelial recovery; fibroblast activation; regulation of matrix turnover	Donor-derived vascular and stromal compartments participate in the local environment in which resolution occurs, while recipient-derived macrophages progressively acquire graft-associated phenotypes	Effective resolution limits structural remodeling; incomplete resolution permits persistent inflammation and fibrosis	Resolution biology is established broadly; its donor–recipient determinants after human HTx remain insufficiently characterized	[108]
Immune–stromal remodeling and fibrosis	Recurrent injury becomes incorporated into the structure of the graft	Macrophage–fibroblast signaling; myofibroblast activation; extracellular-matrix deposition; vascular smooth-muscle-cell modulation; persistent endothelial–immune interactions	Graft-derived stromal and vascular cells provide the structural substrate upon which successive recipient immune responses accumulate	Progressive interstitial fibrosis, vascular remodeling, and increasing susceptibility to further graft injury	Supported by experimental transplantation and human molecular/spatial studies	[105,157]
Establishment of a CAV-specific immune–vascular niche	Chronic allograft injury develops a self-reinforcing vascular microenvironment	Modulated vascular smooth-muscle cells; macrophage subsets; T-cell populations; type I interferon signaling; reciprocal immune–stromal interactions; neointimal matrix accumulation	The mature lesion integrates graft-derived vascular architecture with years of recipient immune pressure and cellular replacement	Persistent vascular inflammation, progressive neointimal expansion, and loss of coronary luminal area	Human single-cell and spatial studies provide direct evidence for distinct immune–stromal programs in CAV	[30]
Clinically established CAV	Cumulative alloimmune, vascular, and reparative injury becomes anatomically and clinically manifest	Diffuse concentric intimal hyperplasia; microvascular dysfunction; chronic endothelial injury; extracellular-matrix accumulation; persistent macrophage and lymphocyte populations	Any initial donor-specific influence is now integrated with recurrent rejection, DSA, infections, immunosuppression, recipient cardiovascular risk factors, and cellular replacement	Progressive ischemia, graft dysfunction, arrhythmia, heart failure, and late graft loss	CAV is well established as a multifactorial late complication; an independent causal contribution of pre-transplant donor immune memory has not been demonstrated	[2,158]

Abbreviations: AMR, antibody-mediated rejection; APC, antigen-presenting cell; CAV, cardiac allograft vasculopathy; DAMP, damage-associated molecular pattern; DSA, donor-specific antibody; HTx, heart transplantation; MHC, major histocompatibility complex; MYD88, myeloid differentiation primary response 88; NK, natural killer; Ref, references; TLR, Toll-like receptor. Note: This continuum integrates established mechanisms of reperfusion-associated innate activation, allorecognition, endothelial injury, inflammatory resolution, immune–stromal remodeling, and CAV with the hypothesis advanced in this review that the pre-existing immune state of donor tissue may modify the subsequent donor–recipient interaction. Evidence currently does not demonstrate that donor-tissue immune memory independently causes rejection or CAV in humans. The proposed framework instead identifies stages at which donor-resident cellular states could be tested as modifiers of recipient-derived alloimmune injury.

**Table 6:** Experimental Approaches to Resolve Donor and Recipient Contributions to the Evolving Cardiac Allograft Immune State.

Experimental Question	Required Sample/Time Point	Preferred Approach	What It Establishes	Principal Limitation	References
What immune states exist before implantation?	Pre-implant donor myocardium	sc/snRNA-seq, immune phenotyping	Baseline cell states	Does not establish persistence	[17]
Is a state spatially restricted?	Pre-implant tissue	Spatial transcriptomics/proteomics	Anatomical niche	Sampling heterogeneity	[6]
Does the state have features of durable programming?	Donor cells/tissue	Epigenomic + functional profiling	Regulatory persistence	Association ≠ memory	[183]
Which cells remain donor-derived?	Serial graft tissue	Genotype-informed single-cell analysis	Cellular provenance	Tissue availability	[16,184]
What states are acquired by recipient cells?	Serial biopsies	Cell-origin-resolved transcriptomics	Cellular succession	Limited biopsy volume	[16,184]
Does the donor state modify recipient immunity?	Paired donor/recipient + longitudinal graft	Interaction modeling	Donor × recipient effect	Requires large prospective cohorts	[17]
Does rejection resolve or persist?	Serial biopsies + blood	Molecular/spatial tissue analysis + dd-cfDNA	Temporal phenotype	Surveillance timing	[185]
Does early biology predict CAV?	Longitudinal cohort	Molecular profiling + vascular imaging	Link to chronic phenotype	Long follow-up/confounding	[186]

Abbreviations: AMR, antibody-mediated rejection; CAV, cardiac allograft vasculopathy; dd-cfDNA, donor-derived cell-free DNA; DSA, donor-specific antibody; EMB, endomyocardial biopsy; HLA, human leukocyte antigen; IFN, interferon; IVUS, intravascular ultrasound; MMDx, Molecular Microscope Diagnostic System; OCT, optical coherence tomography; PGD, primary graft dysfunction; scRNA-seq, single-cell RNA sequencing; snRNA-seq, single-nucleus RNA sequencing. Note: No individual platform can reconstruct the immunological biography of the donor heart in isolation. Pre-implant single-cell, spatial, epigenetic, metabolic and proteomic analyses interrogate complementary components of the donor tissue state, whereas post-transplant dd-cfDNA, molecular biopsy, histopathology and vascular imaging characterize the trajectory that emerges after exposure to recipient immunity. The proposed framework is therefore longitudinal rather than assay-centered. Its clinical value will depend on demonstrating that an integrated donor–recipient phenotype provides information beyond established clinical and immunological risk factors and, importantly, identifies biology that can be modified. At present, these approaches should be regarded primarily as a framework for mechanistic investigation and precision phenotyping; there is insufficient evidence to support donor allocation on the basis of a molecular or immunological donor-heart profile.

## Data Availability

No new data were created or analyzed in this study. Data sharing is not applicable to this article.

## Abbreviations

The following abbreviations are used in this manuscript:

ADP	adenosine diphosphate
AMR	antibody-mediated rejection
APC	antigen-presenting cell
ATP	adenosine triphosphate
C3b	complement component 3b
C5a	complement component 5a
CAV	cardiac allograft vasculopathy
CCR2	C-C chemokine receptor type 2
CRP	C-reactive protein
CXCL5	C-X-C motif chemokine ligand 5
DAMP	damage-associated molecular pattern
dd-cfDNA	donor-derived cell-free DNA
DSA	donor-specific antibody
ECM	extracellular matrix
HLA	human leukocyte antigen
HTx	heart transplantation
ICAM	intercellular adhesion molecule
IFN	interferon
IL-6	interleukin-6
IRI	ischemia–reperfusion injury
JAK	Janus kinase
MHC	major histocompatibility complex
MYD88	myeloid differentiation primary response 88
NK	natural killer
RNA	ribonucleic acid
ROS	reactive oxygen species
SMC	smooth muscle cell
STAT3	signal transducer and activator of transcription 3
TGF-β	transforming growth factor beta
TLR9	Toll-like receptor 9
TNF	tumor necrosis factor
TRM	tissue-resident macrophage
VCAM	vascular cell adhesion molecule
